# MobilePrune: Neural Network Compression via *ℓ*_0_ Sparse Group Lasso on the Mobile System

**DOI:** 10.3390/s22114081

**Published:** 2022-05-27

**Authors:** Yubo Shao, Kaikai Zhao, Zhiwen Cao, Zhehao Peng, Xingang Peng, Pan Li, Yijie Wang, Jianzhu Ma

**Affiliations:** 1 Department of Computer Science, Purdue University, West Lafayette, IN 47907, USA; shao111@purdue.edu (Y.S.); peng272@purdue.edu (Z.P.); panli@purdue.edu (P.L.); 2 Department of Computer Science, Indiana University at Bloomington, Bloomington, IN 47405, USA; kkai_zhao@yeah.net; 3 Department of Computer Graphics, Purdue University, West Lafayette, IN 47907, USA; cao270@purdue.edu; 4 Institute for Interdisciplinary Information Sciences, Tsinghua University, Beijing 100190, China; xingang.peng@gmail.com; 5 Institute for Artificial Intelligence, Peking University, Beijing 100871, China

**Keywords:** mobile computing, model compression, pruning network, deep learning, convolutional neural network

## Abstract

It is hard to directly deploy deep learning models on today’s smartphones due to the substantial computational costs introduced by millions of parameters. To compress the model, we develop an $\ell_{0}$-based sparse group lasso model called MobilePrune which can generate extremely compact neural network models for both desktop and mobile platforms. We adopt group lasso penalty to enforce sparsity at the group level to benefit General Matrix Multiply (GEMM) and develop the very first algorithm that can optimize the $\ell_{0}$ norm in an exact manner and achieve the global convergence guarantee in the deep learning context. MobilePrune also allows complicated group structures to be applied on the group penalty (i.e., trees and overlapping groups) to suit DNN models with more complex architectures. Empirically, we observe the substantial reduction of compression ratio and computational costs for various popular deep learning models on multiple benchmark datasets compared to the state-of-the-art methods. More importantly, the compression models are deployed on the android system to confirm that our approach is able to achieve less response delay and battery consumption on mobile phones.

## 1. Introduction

**Figure 1:**
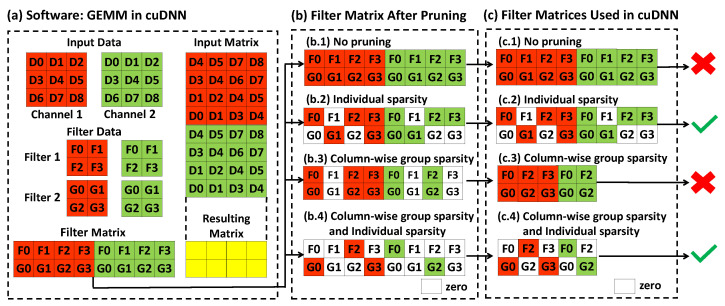
**Observations of different strategies’ pruned filter matrix for hardware acceleration with software implementation of convolution in cuDNN.** (**a**) General Matrix Multiply (GEMM) is applied in cuDNN. (**b**) Different strategies such as no pruning, individual sparsity, column-wise group sparsity, and both individual sparsity and column-wise group sparsity on pruning the filter matrix. (**c**) The pruned filter matrix implemented in cuDNN and determined whether it can be used for hardware acceleration or not.

Deep neural networks (DNNs) have achieved tremendous success in many real-world applications. However, the computational cost of DNN models significantly restricts their deployment on platforms with limited computational resources, such as mobile devices. To address this challenge, numerous model compression algorithms have been proposed to reduce the sizes of DNN models. The most popular solution is to prune the weights with small magnitudes by adding $\ell_{0}$ or $\ell_{1}$ penalties [1,2,3,4]. The non-zero weights selected by these methods are randomly distributed and do not reduce the memory consumption due to the matrix operations widely adopted in nowadays deep learning architectures as shown in Figure 1(b.2). The implementation of such a non-structured sparse matrix in cuDNN [5], which is the Basic Linear Algebra Subroutines library used by deep learning models, has similar memory consumption as the original matrix without pruning, as shown in Figure 1(b.2),(c.2). To overcome the problem, structured pruning models [6,7,8,9] are proposed to enforce group sparsity by pruning a group of pre-defined variables together. By tying the weights connecting to the same neuron together, these approaches are able to prune a number of hidden neurons to reduce the sizes of weight matrices and benefit General Matrix Multiply (GEMM) used in cuDNN as shown in Figure 1(b.3),(c.3). However, one of the main problems of the structured methods is that they do not consider and take advantage of the hardware accelerator architectures.

In this paper, we observe three key factors that could lead to an extremely compact deep network. First, we observe that most modern deep learning architectures rely on the General Matrix Multiply (GEMM) functions implemented in the cuDNN package. We, therefore, propose a new network compression algorithm to harmonize the group selections in the structured penalty and the implementation of GEMM in cuDNN as well as the hardware accelerator using sparse systolic tensor array [10,11]. Figure 1 demonstrates the basic rationale of our observation. In comparison to the pruned model in Figure 1(c.3),(c.4) needs additional sparsity within the remaining groups, which could be utilized by the sparse systolic tensor array for hardware acceleration.

The second observation is that recent studies [12,13] have demonstrated that $\ell_{0}$ norm is the best sparsity-inducing penalty compared to lasso $\ell_{1}$ [14], elastic net [15], SCAD [16], and MCP [17] models. Remarkably, even current $\ell_{0}$ optimization techniques can not achieve the global optimal solution, these $\ell_{0}$-based methods with sub-optimal solutions still significantly outperform other sparsity norms, which can be solved to global optima [13,18]. Hence, the community has shown great interest in using $\ell_{0}$ norm to compress the large-scale DNN models [8,19,20,21,22,23,24]. However, some $\ell_{0}$-based DNN pruning approaches [8,25,26] rely on different relaxation strategies to conquer the non-convex and non-differentiable challenges, which does not fully exploit the strength of $\ell_{0}$ regularization. The other methods [19,20,21,22,23] rely on the alternating direction method of multipliers (ADMM) whose global convergence has not been proved so far when applied to $\ell_{0}$ norm optimization [27,28,29].

Third, most of these algorithms are designed originally for mobile platforms, as the computational resources are relatively rich for desktop applications. However, few of them have been deployed on real mobile systems to test the running time and energy consumption to verify their assumptions.

Therefore, we develop the very first algorithm in this paper, named MobilePrune, which is able to solve the optimization of $\ell_{0}$ sparse group lasso regularization in an exact manner. The main technical contribution is that we solve the proximal operators for $\ell_{0}$ sparse group lasso, where groups in the group lasso term could have overlapping group structure and tree structure [30]. From the theoretical point of view, we prove our algorithm always converges to the critical point (local optimal point) under mild conditions. In addition, we conduct extensive experiments on multiple public datasets and find MobilePrune achieves superior performance at sparsifying networks with both fully connected and convolutional layers. More importantly, we deploy our system on the real android system on several mobile devices and test the performance of the algorithm on multiple Human Activity Recognition (HAR) tasks. The results show that MobilePrune achieves much lower energy consumption and higher pruning rate while still retaining high prediction accuracy. Besides a powerful network compression algorithm, this work also provides a valuable platform and mobile dataset for further work to evaluate their methods in a very real scenario.

The rest of this paper is organized as follows. Section 2 provides the relevant background and related work. In Section 3, we give a brief overview of the proposed MobilePrune methods. In Section 4, we discuss the detailed information of the proposed methods and algorithms. In Section 5, we describe how the experiments are set up and evaluate the proposed methods from different perspectives. Section 6 discusses the future work and summarizes the paper.

## 2. Related Work

### 2.1. Sparsity for Deep Learning Models

Many pruning algorithms for deep learning models achieve slim neural networks by introducing sparse-inducing norms to the models. $\ell_{1}$ regularization [31,32,33] and $\ell_{0}$ regularization [8] were applied to induce sparsity on each individual weight. However, such individual sparsity has arbitrary structures, which cannot be utilized by software and hardware. Wei et al. [24] applied group sparsity to prune filters or channels, which can reduce the matrix size used in GEMM in cuDNN. Because the pruned models are compatible with cuDNN, they achieved large speedups. There are methods [34,35] aiming to find sparse models at both individual and group levels, which is similar to our goal. However, they all used $\ell_{1}$ norm to induce individual sparsity in addition to group sparsity. We have performed a comprehensive comparison and demonstrated that our MobilePrune method is the best in inducing sparsity at both individual and group levels for pruning deep learning models in Section 5.

### 2.2. Learning Algorithms for $\ell_{0}$ Norm

Recent studies [12,13] demonstrate that $\ell_{0}$ norm is the best sparsity-inducing penalty comparing to lasso $\ell_{1}$ [14], elastic net [15], SCAD [16], and MCP [17] models. Remarkably, even current $\ell_{0}$ optimization techniques cannot achieve the global optimal solution, these $\ell_{0}$-based methods with sub-optimal solutions still significantly outperform $\ell_{1}$ and elastic net models, which can be solved to global optima [13,18]. Therefore, the machine learning community has shown great interest in using $\ell_{0}$ norm to compress the large-scale DNN models [8,19,20,21,22,23,24]. However, some $\ell_{0}$-based DNN pruning approaches [8,25] rely on different relaxation strategies to conquer the non-convex and non-differentiable challenges, which does not fully exploit the strength of $\ell_{0}$ regularization. The other methods [19,20,21,22,23] rely on the alternating direction method of multipliers (ADMM) whose global convergence has not proved so far when applied to $\ell_{0}$ norm optimization [27,28,29].

### 2.3. Software & Hardware Compatibility

In this paper, we aim to design an algorithm to make the pruned DNN models compatible with cuDNN library [5] and hardware accelerator architecture that uses the sparse systolic tensor array [11,36]. cuDNN is the GPU-accelerated library used in deep learning models [5]. As shown in Figure 1a, convolution used in the convolutional neural network is lowered to matrix multiplication. Therefore, the size of the filter matrix can be reduced when inducing group sparsity column-wise as shown in Figure 1(b.3),(b.4), which can reduce the memory of the DNN models to achieve practical performance improvement. The systolic tensor array is an efficient hardware accelerator for structured sparse matrix as shown in Figure 1(c.4). Specifically, each column in Figure 1(c.4) is sparse. To achieve a pruned DNN model that is compatible with cuDNN and the systolic tensor array, sparsity needs to be induced on both the group level and within-group level. We will show how we achieve this in the following sections.

## 3. Overview

**Figure 2:**
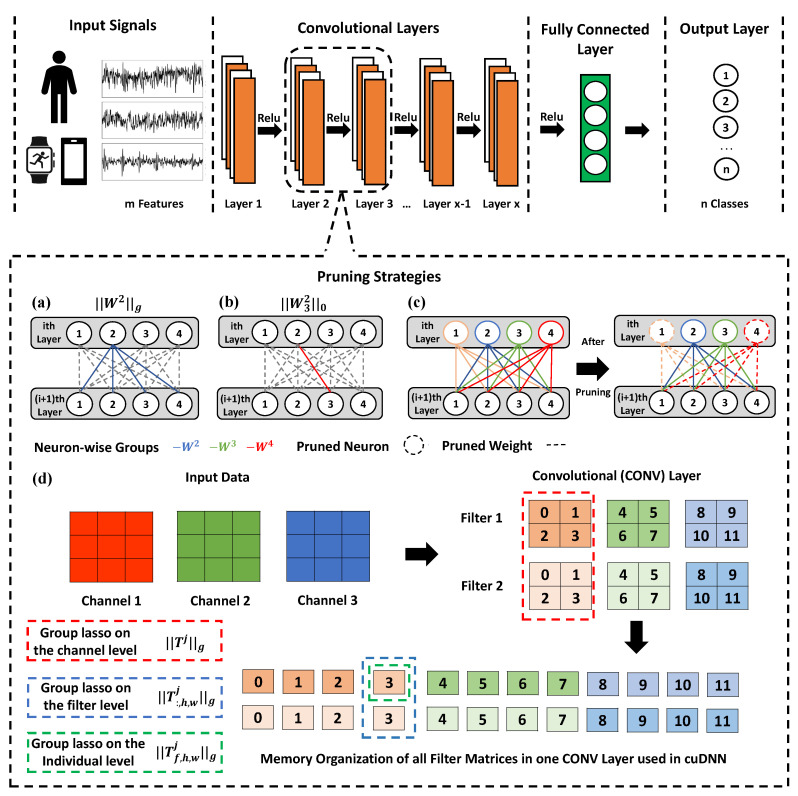
**Overview of the proposed MobilePrune method.** (**a**) Group sparsity for weights of a neuron for fully connected layers. (**b**) Sparsity on individual weights for fully connected layers. (**c**) Pruning strategy for fully connected layers and their effect where sparsity is induced on both neuron-wise groups and individual weights. (**d**) Group and individual sparsity for convolutional layers.

The central idea of MobilePrune is to compress the deep learning model in a way that is compatible with the architecture of data organization in the memory by combining $\ell_{0}$ regularization and group lasso regularization. The group lasso regularization helps to keep important groups of weights that benefit cuDNN, while $\ell_{0}$ regularization helps to achieve additional sparsity within those important groups that are needed for hardware acceleration. Figure 2 provides an overview of the proposed MobilePrune method. As illustrated in Figure 2a–c, the group lasso penalty will remove all the weights together with the *i*th neuron if it is less important for the prediction and if a group is selected, the $\ell_{0}$ penalty further removes the weights with small magnitudes within the group. Note that zeroing out the weights connected to the *i*th neuron results in removing the *i*th neuron and all the associated weights entirely. We will discuss more detail information in next section.

## 4. Methods

Our main objective is to obtain a sparse deep neural network with a significantly less number of parameters at both individual and group levels by using the proposed novel combined regularizer: $\ell_{0}$ sparse group lasso.

### 4.1. $\ell_{0}$ Sparse Group Lasso

We aim to prune a generic (deep) neural network, which includes fully connected feed-forward networks (FCN) and convolutional neural networks (CNN). Assuming the generic neural network has *N* neurons in FCN and *M* channels in CNN. Let $W^{i}$ denote the outgoing weights of the *i*th neuron in FCN and $T^{j}$ represent the 3D tensor of all filters in the *j*th channel, which can come from different layers. The training objective for the neural network is given as follows:

(1) $$\begin{array}{cc} \min_{W,T}:L\left(W,T;D\right) & +\Omega_{\lambda }^{\eta }\left(W\right)+\Gamma_{\beta ,\gamma }^{\alpha }\left(T\right) \end{array}$$

where $W=\{W^{1},\ldots ,W^{N}\}$, $T=\{T^{1},\ldots ,T^{M}\}$, $D={\left\{x_{i},y_{i}\right\}}_{i=1}^{P}$ is a training dataset with *P* instances, L is an arbitrary loss function parameteized by *W* and *T*, $\Omega_{\lambda }^{\eta }\left(W\right)$ and $\Gamma_{\beta ,\gamma }^{\alpha }\left(T\right)$ represent the $\ell_{0}$ sparse group lasso penalties for neurons and channels, respectively. Specifically, $\Omega_{\lambda }^{\eta }\left(W\right)$ is defined as

(2) $$\begin{array}{c} \Omega_{\lambda }^{\eta }\left(W\right)=\sum_{i=1}^{N}(\eta ∥W^{i}{∥}_{0}+\lambda ∥W^{i}{∥}_{g}) \end{array}$$

where η≥0 and λ≥0 are regularization parameters. Let n(i) represent the set of outgoing edge weights of neuron *i*. Then, $∥W^{i}{∥}_{0}=\sum_{j\in n(i)}{∥W_{j}^{i}∥}_{0}$ ($W_{j}^{i}$ is the *j*th outgoing edge weight of neuron *i*; $∥W_{j}^{i}{∥}_{0}=0$ when $W_{j}^{i}=0$ and $∥W_{j}^{i}{∥}_{0}=1$ otherwise) computes the number of the non-zero edges in $W^{i}$ and $∥W^{i}{∥}_{g}=\sqrt{\sum_{j}{\left(W_{j\in n(i)}^{i}\right)}^{2}}$ aggregates the weights associated with the *i*th neuron as a group. The core spirit of Equation (2) is illustrated in Figure 2a–c, the group lasso penalty $∥W^{i}{∥}_{g}$ tends to remove all the weights together with the *i*th neuron if it is less important. If a group is selected, the $\ell_{0}$ penalty further removes the weights with small magnitudes within the group. Group sparsity $∥W^{i}{∥}_{g}$ can help remove neurons in the neural network, which reduces the size of the neural network and further improve efficiency. Individual sparsity $∥W^{i}{∥}_{0}$ helps to achieve additional sparsity within the remaining neurons. Such structured sparsity can be used by the systolic tensor array [10,11]. The other regularization term $\Gamma_{\beta ,\gamma }^{\alpha }\left(T\right)$ is defined as following,

(3) $$\begin{array}{c} \Gamma_{\beta ,\gamma }^{\alpha }\left(T\right)=\sum_{j=1}^{M}(\alpha ∥T^{j}{∥}_{0}+\beta ∥T^{j}{∥}_{g}+\gamma \sum_{h,w}∥T_{:,h,w}^{j}{∥}_{g}), \end{array}$$

where α,β, and γ are non-negative regularization parameters. Equation (3) defines a hierarchical-structured sparse penalty in which structure is guided by the memory organization of GEMM using cuDNN [5]. As demonstrated in Figure 2d, the pruning strategy encoded in Equation (3) explicitly takes advantage of the GEMM used in cuDNN. $∥T^{j}{∥}_{g}$ enforces the group sparsity of all the filters applied to the *j*th channel and $∥T_{:,h,w}^{j}{∥}_{g}$ enforces the group sparsity across the filters on the same channel and $∥T^{j}{∥}_{0}=\sum_{f}\sum_{h}\sum_{w}{∥T_{f,h,w}∥}_{0}$ prunes the small weights within the remaining channels and filters. Equation (3) can help to achieve an extremely compact model as Figure 1(c.4). Therefore, the computation can be accelerated at both software and hardware levels.

### 4.2. Exact Optimization by PALM

In this subsection, we first briefly review the general PALM (Proximal Alternating Linearized Minimization) framework used in our MobilePrune algorithm. Then, we introduce how we modify PALM to efficiently optimize the $\ell_{0}$ sparse group lasso for neural network compression. PALM is designed to optimize a general optimization problem formulated as:

(4) $$\begin{array}{c} \min_{W,T}:F\left(W,T\right)+\Phi_{1}\left(W\right)+\Phi_{2}\left(T\right). \end{array}$$

where F(W,T) is a smooth function and $\Phi_{1}\left(W\right)$ and $\Phi_{2}\left(T\right)$ do not need to be convex or smooth, but are required to be lower semi-continuous. The PALM algorithm applies proximal forward–backward algorithm [37] to optimize Equation (4) with respect to *W* and *T* in an alternative manner. Specifically, at iteration k+1, the temporal values of $W^{\left(k+1\right)}$ and $T^{\left(k+1\right)}$ for the proximal forward–backward mapping are derived by solving the following sub-problems,

(5) $$\begin{array}{cc} W^{\left(k+1\right)} & \in \min_{W}:\left\{\frac{c^{k}}{2}{∥W-U_{i}^{\left(k\right)}∥}_{F}^{2}+\Phi_{1}\left(W\right)\right\}, \end{array}$$

(6) $$\begin{array}{cc} T^{\left(k+1\right)} & \in \min_{T}:\left\{\frac{d^{k}}{2}{∥T-V_{i}^{\left(k\right)}∥}_{F}^{2}+\Phi_{2}\left(T\right)\right\}, \end{array}$$

where $U_{i}^{\left(k+1\right)}=W_{i}^{\left(k\right)}-\frac{1}{c^{k}}\nabla_{W}F\left(W^{\left(k\right)},T^{\left(k\right)}\right)$ and $V_{i}^{\left(k+1\right)}=T_{i}^{\left(k\right)}-\frac{1}{d^{k}}\nabla_{T}F\left(W^{\left(k+1\right)},T^{\left(k\right)}\right)$. Additionally, $c^{k}$ and $d^{k}$ are positive constants. This optimization process has been proven to converge to a critical point when functions *F*, $\Phi_{1}$, and $\Phi_{2}$ are bounded [37]. We further extend the convergence proof in [37] and prove that the global convergence of PALM holds for training deep learning models under mild conditions. The detailed proof can be found in Appendix A.

To optimize Equation (1), we define two proximal operators for the two penalty terms as the following,

(7) $$\begin{array}{cc} \pi_{\lambda }^{\eta }\left(y\right) & \equiv \arg\min_{x}\left\{\frac{1}{2}{∥x-y∥}_{2}^{2}+\Omega_{\lambda }^{\eta }\left(x\right)\right\}, \end{array}$$

(8) $$\begin{array}{cc} \theta_{\beta ,\gamma }^{\alpha }\left(y\right) & \equiv \arg\min_{x}\left\{\frac{1}{2}{∥x-y∥}_{2}^{2}+\Gamma_{\beta ,\gamma }^{\alpha }\left(x\right)\right\}. \end{array}$$

Here, functions $\Omega_{\lambda }^{\eta }\left(\cdot \right)$ and $\Gamma_{\beta ,\lambda }^{\alpha }\left(\cdot \right)$ take vectors as inputs, which are equivalent to Equations (2) and (3) once we vectorize $W^{i}$ and $T^{j}$. The overall optimization process of MobilePrune is described in Algorithm 1. Once we can efficiently compute the optimal solution of $\pi_{\lambda }^{\eta }\left(\cdot \right)$ and $\theta_{\beta ,\lambda }^{\alpha }\left(\cdot \right)$, the computational burden mainly concentrates on the partial derivative calculation of functions H(·) and F(·), which is the same as training a normal DNN model.
**Algorithm 1** The framework of MobilePrune Algorithm.Initialize ${\left(W^{i}\right)}^{0},\forall i$, ${\left(T^{j}\right)}^{0},\forall j$ and $L_{r}$.**for**k=0,2,…**do**   **for** i=1,2,…,N **do**     $H_{k}^{i}=\nabla_{W_{k}^{i}}L\left(W_{k}^{i},W_{k}^{j\neq i},T_{k}\right)$.     $W_{k+1}^{i}\in \pi_{\lambda /L_{r}}^{\eta /L_{r}}\left(W_{k}^{i}-\frac{1}{L_{r}}H_{k}^{i}\right)$ by solving Equation (7).   **end for**   **for** j=1,2,…,M **do**     $F_{k}^{j}=\nabla_{T_{k}^{j}}L\left(W_{k+1},T_{k}^{j},T_{k}^{l\neq j}\right)$.     $T_{k+1}^{j}\in \theta_{\beta /L_{r},\gamma /L_{r}}^{\alpha /L_{r}}\left(T_{k}^{j}-\frac{1}{L_{r}}F_{k}^{j}\right)$ by solving Equation (8).   **end for****end for**

### 4.3. Efficient Computation of Proximal Operators

To the best of our knowledge, $\pi_{\lambda }^{\eta }\left(y\right)$ and $\theta_{\beta ,\gamma }^{\alpha }\left(y\right)$ defined in Equations (7) and (8) are novel proximal operators that have not been attempted before. Solving $\pi_{\lambda }^{\eta }\left(y\right)$ and $\theta_{\beta ,\gamma }^{\alpha }\left(y\right)$ is the key to apply MobilePrune in Algorithm 1. Therefore, this subsection elaborates the algorithmic contributions we made to efficiently calculate $\pi_{\lambda }^{\eta }\left(y\right)$ and $\theta_{\beta ,\gamma }^{\alpha }\left(y\right)$.

#### 4.3.1. Proximal Operator $\pi_{\lambda }^{\eta }\left(\cdot \right)$

The difficulty of solving $\pi_{\lambda }^{\eta }\left(y\right)$ in Equation (9) is that both ${∥x∥}_{g}$ and ${∥x∥}_{0}$ are not differentiable when the vector x=0. Furthermore, ${∥x∥}_{0}$ calculates the number of non-zeros in the vector $x\in R^{n}$ and there are $C\left(n,0\right)+C\left(n,1\right)+\ldots +C\left(n,n\right)=2^{n}$ (C(n,k) computes the number of non-zero patterns in *x* where *k* elements in *x* are not zeros) possible combinations, which indicates the brute-force method needs $2^{n}$ computations to find the global optimal solution. However, here we prove that the $\pi_{\lambda }^{\eta }\left(y\right)$ in Equation (9) can be efficiently solved by a O(nlog(n)) algorithm in a closed from. We illustrate the algorithm in Algorithm 2 and prove its correctness in Theorem 1. To the best of our knowledge, it is the first efficient algorithm that can calculate this novel proximal operator.

**Theorem** **1.** *The proximal operator $\pi_{\lambda }^{\eta }\left(y\right)$ can be written as*

(9) $$\begin{array}{c} \pi_{\lambda }^{\eta }\left(y\right)\equiv \arg\min_{x}\left\{f\left(x\right):=\frac{1}{2}{∥x-y∥}_{2}^{2}+{\lambda ∥x∥}_{g}+\eta {∥x∥}_{0}\right\} \end{array}$$

*The optimal solution of this proximal operator can be computed by Algorithm 2.*

**Algorithm 2** Efficient calculation of $\pi_{\lambda }^{\eta }\left(y\right)$
**Input:** A sorted vector *y*, such that $|y_{1}\left|\geq \right|y_{2}|\geq \ldots$
**Output:** $x^{*}$


**for** i=0,…,n **do**
   **if** $∥\vec{y_{i}}{∥}_{g}\leq \lambda$ **then**     $U_{i}=\frac{1}{2}{∥y∥}_{2}^{2}$   **else**     $U_{i}=-\frac{1}{2}(∥\vec{y_{i}}{∥}_{g}{-\lambda )}^{2}+i\eta +\frac{1}{2}{∥y∥}_{2}^{2}$   **end if**
**end for**
Compute $k=\arg\min_{j}U_{j}$
**if** $U_{k}\geq \frac{1}{2}{∥y∥}_{2}^{2}$ **then**
   $x^{*}=0$
**else**
   $x^{*}=(∥\vec{y_{k}}{∥}_{g}-\lambda )\frac{\vec{y_{k}}}{∥\vec{y_{k}}{∥}_{g}}$
**end if**

**Proof.**  Without loss of generality, we assume $y={\left[y_{1},y_{2},\ldots ,y_{n}\right]}^{T}\in R^{n}$ is an ordered vector, where $|y_{1}\left|\geq \right|y_{2}\left|\geq \ldots \geq \right|y_{n}|$. Then, we define $\vec{y_{k}}=\left[y_{1},y_{2},\ldots ,y_{k},0,\ldots ,0\right]$, where the top *k* elements with largest absolute values are kept and all the rest elements are set to zeroes. We define another set $\Phi^{k}={\{x|∥x∥}_{0}=k,x\in R^{n}\}$ to represent all *n*-dimensional vectors with exact *k* non-zero elements. For any $x={\left[x_{1},x_{2},\ldots ,x_{n}\right]}^{T}\in \Phi^{k}$, we further define a mask function $e:e_{i}=1\left\{x_{i}=0\right\}$ to reveal the non-zero locations of *x*.

Since we do not know how many non-zero elements remain in the optimal solution of Equation (9), we need to enumerate all possible *k* and solve n+1 sub-problems for all $x^{k}\in \Phi^{k}$ with k=0,…,n. For each *k*, the sub-problem is defined as

(10) $$\begin{array}{cc} \; & \min_{x^{k}\in \Phi^{k}}f\left(x^{k}\right):=\frac{1}{2}{∥x^{k}-y∥}_{2}^{2}+\Omega_{\lambda }^{\eta }\left(x^{k}\right)\\ \Leftrightarrow & \min_{x^{k}\in \Phi^{k}}\frac{1}{2}∥x^{k}-y^{k}{∥}_{2}^{2}+\frac{1}{2}{∥\bar{y^{k}}∥}_{2}^{2}+\Omega_{\lambda }^{\eta }\left(x^{k}\right) \end{array}$$

Based on Lemma A4 in Appendix A.2, we observe that if $∥y^{k}{∥}_{g}\leq \lambda$, then $x^{k*}=0$ and $f\left(x^{k*}\right)=\frac{1}{2}{∥y∥}_{2}^{2}$. If $∥y^{k}{∥}_{g}>\lambda$, then $x^{k*}=(∥y^{k}{∥}_{g}-\lambda )\frac{y^{k}}{∥y^{k}{∥}_{g}}$ and the value of the objective function can be computed as

(11) $$\begin{array}{cc} f(x^{k*})= & \frac{1}{2}∥x^{k*}-y^{k}{∥}_{2}^{2}+\frac{1}{2}{∥\bar{y^{k}}∥}_{2}^{2}+\Omega_{\lambda }^{\eta }\left(x^{k*}\right)\\ = & -\frac{1}{2}(∥y^{k}{∥}_{g}{-\lambda )}^{2}+\eta k+\frac{1}{2}{∥y∥}_{2}^{2}. \end{array}$$

Equation (11) tells us $f(x^{k*})$ is a function of $y^{k}$. The task of calculating the minimum value of function $f(x^{k*})$ is transformed into solving another optimization $-\frac{1}{2}(∥y^{k}{∥}_{g}{-\lambda )}^{2}+\eta k+\frac{1}{2}{∥y∥}_{2}^{2}$ with respect to $y^{k}$, which is equivalent to ask which of the *k* components of *y* can achieve the minimum value of *f*. Since we assume $∥y^{k}{∥}_{g}>\lambda$, the optimal solution is clearly to select the top *k* components with the largest value from *y*. Therefore we have $\vec{y_{k}}={arg min}_{y^{k}}-\frac{1}{2}(∥y^{k}{∥}_{g}{-\lambda )}^{2}+\eta k+\frac{1}{2}{∥y∥}_{2}^{2}$ and the corresponding $x^{k*}=(∥\vec{y_{k}}{∥}_{g}-\lambda )\frac{\vec{y_{k}}}{∥\vec{y_{k}}{∥}_{g}}$. Furthermore, the objective function value is $-\frac{1}{2}(∥\vec{y_{k}}{∥}_{g}{-\lambda )}^{2}+\eta k+\frac{1}{2}{∥y∥}_{2}^{2}$. Hence, problem (10) has a closed-form solution.    □

As shown in Algorithm 2, the heaviest computation is to sort the input vector *y*, therefore, the time complexity for solving Equation (9) is O(nlog(n)).

#### 4.3.2. Proximal Operator $\theta_{\beta ,\gamma }^{\alpha }\left(y\right)$

Similar as $\pi_{\lambda }^{\eta }\left(y\right)$, $\theta_{\beta ,\gamma }^{\alpha }\left(y\right)$ is the solution of the following optimization problem:

(12) $$\begin{array}{cc} \min_{x}: & \kappa \left(x\right):=\frac{1}{2}{∥x-y∥}_{2}^{2}+{\alpha ∥x∥}_{0}+{\beta ∥x∥}_{g}+\gamma \sum_{i=1}^{d}{∥x_{G_{i}}∥}_{g}\\ s.t. & \;\;\overset{d}{\underset{i=1}{⋃}}G_{i}=\left\{1,2,\ldots ,n\right\},G_{i}\cap G_{j}=\emptyset ,\;\forall i,j \end{array}$$

where we assume $x,y\in R^{n}$. $G_{i}\subseteq \left\{1,\ldots ,n\right\},i$ is the index of a group and *d* represents the number of groups. Note that the grouping structures specified in Equation (12) is a special case of the grouped tree structures [30], where ${∥x∥}_{g}$ is the group lasso for the root of the tree and all the $∥x_{G_{i}}{∥}_{g}$ are the group lasso terms of its children. Notice that groups from the same depth on the tree do not overlap and furthermore ${∥x∥}_{0}=\sum_{i=1}^{d}{∥x_{G_{i}}∥}_{0}$. To simplify the notation, assuming ${∥x∥}_{0}\neq 0$ we define $h\left(x\right)=\frac{1}{2}{∥x-y∥}_{2}^{2}+\beta {∥x∥}_{g}$ that is a convex and differentiable and rewrite the problem as

(13) $$\begin{array}{cc} \min_{x}:\;\; & h\left(x\right)+\sum_{i=1}^{d}\Omega_{\gamma }^{\alpha }\left(x_{G_{i}}\right)\\ s.t.\;\; & x\neq 0,\overset{d}{\underset{i=1}{⋃}}G_{i}=\left\{1,2,\ldots ,n\right\},G_{i}\cap G_{j}=\emptyset ,\;\forall i,j\in \left\{1,\ldots ,d\right\}, \end{array}$$

where $\sum_{i=1}^{d}\Omega_{\gamma }^{\alpha }\left(x_{G_{i}}\right)=\sum_{i=1}^{d}\alpha ∥x_{G_{i}}{∥}_{0}+\gamma {∥x_{G_{i}}∥}_{g}$. We can use the proximal method [38] to find a solution $x^{†}$ of Equation (13). In the proximal method, we need to estimate the Lipschitz constant $L\left(x\right)=1+\frac{\beta }{{∥x∥}_{g}}$ and the partial derivative $\nabla_{x_{G_{i}}}h\left(x\right)=\left(1+\frac{\beta }{{∥x∥}_{g}}\right)x_{G_{i}}-y_{G_{i}}$. In addition, we need to use Algorithm 2 to solve $\pi_{\gamma }^{\alpha }\left(\cdot \right)$ for each group $x_{G_{i}}$. After obtaining $x^{†}$, we can find the solution of Equation (12) by comparing κ(0) with $\kappa (x^{†})$. If $\kappa \left(0\right)\leq \kappa \left(x^{†}\right)$, then the local optimal solution of Equation (12) is $x^{*}=0$, otherwise, $x^{*}=x^{†}$. We elaborate the algorithm for the proximal operator $\theta_{\beta ,\gamma }^{\alpha }\left(y\right)$ in the Algorithm 3. The major computation cost is the proximal method, therefore, the convergence rate of Algorithm 3 is O(1/k) [38].
**Algorithm 3** Efficient calculation of $\theta_{\beta ,\gamma }^{\alpha }\left(y\right)$**Input:**$L^{0}$ and $x^{0}$**Output:**$x^{*}$**for**l=0,1,2,…,k**do**   Let $L^{l}=1+\frac{\beta }{∥x^{l}{∥}_{g}}$ and $u=x_{G_{i}}^{l}-\frac{1}{L^{l}}\nabla_{x_{G_{i}}}h\left(x^{l}\right)$, then compute $x_{G_{i}}^{l}\in \pi_{\gamma /L^{l}}^{\alpha /L^{l}}\left(u\right),\;\forall i$ by applying Algorithm 2.**end for****if**$\kappa \left(0\right)\leq \kappa \left(x^{k}\right)$**then**   $x^{*}=0$**else**   $x^{*}=x^{k}$**end if**

## 5. Experimental Setup and Results

### 5.1. Performance on Image Benchmarks

In this subsection, we compared our proposed MobilePrune approach with other state-of-the-art pruning methods in terms of prune rate, computational costs, and test accuracy. We mainly compared our methods with structured pruning methods because DNN models pruned by non-structure pruning methods could not obtain practical speedup as shown in Figure 1. Notably, we only compared the results that can be reproduced by the source codes provided by the competing methods. First, we briefly summarized their methodology. PF [32] and NN slimming [33] were simple magnitude-based pruning methods based on l1 norm. BC [9], SBP [7], and VIBNet [39] cast the DNN pruning into probabilistic Bayesian models. C-OBD [40], C-OBS [2], Kron-OBD [40,41], Kron-OBS [2,41], and EigenDamage [42] are Hessian matrix-based methods. $\ell_{0}$ norm penalized method [8] and group lasso penalized method [24] are also well-known methods.

In our experiments, we use NVIDIA Corporation as the GPU and the number of cores of the CPU is 12. All the baseline models were trained from scratch via stochastic gradient decent(SGD) with a momentum of 0.9. We trained the networks for 150 epochs on MNIST and 400 epochs on CIFAR-10 and Tiny-ImageNet with an initial learning rate of 0.1 and weight decay of 5 × 10^−4^. The learning rate is decayed by a factor of 10 at 50, 100 on MNIST and at 100, 200 on CIFAR-10 and Tiny-ImageNet, respectively. The details of hyper-parameters for all experiments are summarized in Appendix B. We also provide the computational efficiency of our methods in Appendix C.

#### 5.1.1. MNIST Dataset

We first applied MobilePrune to prune the LeNet-300-100 and LeNet-5 [7,8,9] models on the MNIST dataset [43]. LeNet-300-100 is a fully-connected neural network model with three layers and 267 K parameters. LeNet-5 is comprised of two [20, 50] convolutional layers and two [800, 500] fully-connected layers with 431K parameters. Here, we compared with the state-of-the-art structured network compression algorithms [7,8,9] in terms of pruned accuracy, remaining parameters, pruned architecture, and FLOPs of the pruned models.

As shown in the top half of the MNIST dataset of Table 1, our model achieves the least number of neurons after pruning the LeNet-300-100 model and the lowest drop of the prediction accuracy 0.01% compared to other methods. More importantly, our pruned model achieves the lowest FLOPs. Note that the architecture of our pruned model is as compact as L0-sep [8], but is extremely sparse with only 5252 weights left. This additional sparsity would be critical when applying hardware acceleration [10,11] to our pruned model.

In addition, we compared with SSL on pruning the first two convolutional layers as done in [24] in Table A2. SSL has the same group lasso penalty term as ours but without $\ell_{0}$ norm regularization. As shown, our method decreases the sizes of the filters from 25 and 500 to 16 and 65, respectively, which dramatically lowers the FLOPs. In addition, the non-zero parameters in those remaining filters is very sparse in our model.

The bottom half of the MNIST dataset in Table 1 shows the performance comparison on pruning the LeNet-5 model. The LeNet-5 model pruned by our method achieves the lowest FLOPs (113.50 K) with the smallest predicting accuracy drop 0.01%. Moreover, our pruned model also has the smallest number of weights (around 2310). In addition, we compared with SSL on pruning the first two convolutional layers as done in [24]. SSL has the same group lasso penalty term as ours, but without $\ell_{0}$ norm regularization. More details about SSL can be found in Appendix C.2. As shown, our method decreases the sizes of the filters from 25 and 500 to 16 and 65, respectively, which dramatically lowers the FLOPs. In addition, the non-zero parameters in those remaining filters are very sparse in our model.

#### 5.1.2. CIFAR-10 Dataset

We further evaluated our method on pruning more sophisticated DNN architectures, VGG-like [44] and ResNet-32 [42,45] and widen the network by a factor of 4, on CIFAR-10 [46]. Similarly, we compared with the state-of-the-art structured pruning methods [2,7,32,39,40,41,42] in terms of various metrics. As shown in the middle of Table 1, the pruned VGG-like model obtained by our method achieves the lowest FLOPs with the smallest test accuracy drop. Similar to previous results, our pruned model is able to keep the smallest number of weights in comparison to other methods, the key for hardware acceleration [10,11]. As presented in Table 1, the pruned ResNet-32 model achieved by our method outperforms other pruned models in terms of pruned test accuracy and FLOPs. In addition, in terms of the remaining weights, our pruned model is at the same sparsity level as C-OBD [40] while our pruned accuracy outperforms C-OBD by a large margin.

#### 5.1.3. Tiny-ImageNet Dataset

Besides the experiments on MNIST and CIFAR-10 datasets, we further evaluated the performance of our method on a more complex dataset, Tiny-ImageNet [47], using VGG-19 [48]. Tiny-ImageNet is a subset of the full ImageNet, which consists of 100,000 images for validation. There are 200 classes in Tiny-ImageNet. We compared our method with some state-of-the-art methods [2,33,40,42] in Table 1. As shown in Table 1, the test accuracy of the pruned model derived from our method outperforms all the other methods by a significant margin, about 10%, except EigenDamage. Our proposed method obtains the same-level test accuracy as EigenDamage. However, our method achieves a much sparser DNN model with 1.16 million fewer weights than EigenDamage. Meanwhile, our pruned model achieves lower FLOPs.

### 5.2. Performance on Human Activity Recognition Benchmarks

To demonstrate the efficacy and effectiveness of our proposed MobilePrune method, we perform a series of compassion studies with other state-of-the-art pruning methods such as $\ell_{0}$ norm, $\ell_{1}$ norm, $\ell_{2}$ norm, group lasso, and $\ell_{1}$ sparse group lasso for all three datasets—WISDM [49,50], UCI-HAR [51,52], and PAMAP2 [53,54,55]. We evaluate the pruning accuracy and pruning rate of weights (parameters) and nodes for our proposed MobilePrune approach and all other state-of-the-art pruning methods using the same learning rate (0.0001) and the same number of epochs (150) for all three datasets. The pruning thresholds are 0.015, 0.005, 0.01 for the pruning methods in the WISDM, HCI-HAR, and PAMAP2 datasets, respectively. In addition, we evaluate the computational cost and battery consumption for our proposed method with all other state-of-the-art pruning methods as well. The details of the dataset descriptions and the hyper-parameters for all experiments are summarized in Appendix D.

#### 5.2.1. Performance on the Desktop

We use Google Colab [56] to build a PyTorch backend on the above datasets. The GPU for Google Colab is NVIDIA Tesla K80 and the number of cores of the CPU for Google Colab is 2. As shown in Table 2, if we only use $\ell_{0}$ norm penalty or $\ell_{2}$ norm penalty, there is no effect on neurons or channels pruning as expected for all three datasets. Similarly, if we only employ group lasso penalty, the pruned model still has more weights or nodes left. For the UCI-HAR dataset, $\ell_{1}$ norm penalty and $\ell_{1}$ sparse group lasso penalty cannot sparse down the model while for the other two datasets, these two penalties could achieve better sparsity, but cannot be better than MobilePrune approach. There exists a trade-off between the pruned accuracy and the pruning rate. As can be seen in Table 2, our MobilePrune method still has high pruned accuracy even if there are not too many parameters and nodes left. In addition, we compare our MobilePrune method with $\ell_{1}$ sparse group lasso penalty. The $\ell_{0}$ sparse group lasso model still significantly outperforms the $\ell_{1}$ sparse group lasso model in weights and nodes pruning, which demonstrates its superiority in pruning CNN models.

We also calculate the response delay and time saving percentage for all the above methods on the desktop platform. Response delay is the time needed for the desktop to run the pre-trained model after the raw input signal is ready. Here in Table 2, the response delay results are obtained after running 200 input samples. As can be seen in Table 2, MobilePrune could save up to 66.00%, 57.43%, 90.20% on response delay on WISDM, HCI-HAR, and PAMAP2 datasets, respectively.

Overall, if we apply MobilePrune method, the pruned CNN models can still achieve the best sparsity in terms of both neurons (or channel) and weights without loss of performance. Additionally, the results in Table 2 show that our MobilePrune method could achieve 28.03%, 46.83%, 3.72% on weight (parameter) sparsity, and 52.52%, 68.75%, and 10.74% on node sparsity for the WISDM, UCI-HAR, and PAMAP2 datasets, respectively.

#### 5.2.2. Performance of Mobile Phones

We evaluate the computational cost and battery consumption for our proposed MobilePrune approach with all other state-of-the-art pruning methods. In order to obtain the final results about how these models perform on today’s smartphone, we implement an Android Application using Android Studio on Huawei P20 and OnePlus 8 Pro. PyTorch Android API [57] is used here for running trained models on Android devices. Currently, the Android devices only support running machine learning models by using CPU only. For the Huawei P20, the CPU is Cortex-A73. For the OnePlus 8 Pro, it is using Octa-Core as its CPU. We also use the Batterystats tool and the Battery Historian script [58] to test the battery consumption.

Table 3 shows the response delay results and battery usage for our proposed method and all other state-to-the-arts pruning methods. Response delay is the time needed for the smartphone’s system to run the pre-trained model after the raw input signal is ready. Here, in Table 3, the response delay results are obtained after running 200 input samples and the battery consumption results are obtained after running 2000 input samples for each penalty in all three datasets. For the HCI-HAR dataset, our MobilePrune approach could save up to 40.14%, 22.22% on response delay and 34.52%, 19.44% on battery usage for Huawei P20 and OnePlus Pro 8, respectively, while the other pruning methods stay almost the same compared to the uncompressed version. For the WISDM and PAMAP2 datasets, $\ell_{0}$ norm penalty, $\ell_{2}$ norm penalty, and group lasso penalty cannot sparse down the model, and therefore they cannot provide any savings in both response delay and battery consumption. $\ell_{1}$ norm and $\ell_{1}$ sparse group lasso methods could provide better time saving and battery consumption saving compared to those three penalties, but they still cannot perform better than the MobilePrune method, which saves 61.94% and 88.15% in response time, and 37.50% and 36.71% in battery consumption for WISDM and PAMAP2 dataset, respectively, on Huawei P20. Additionally, it also saves 52.08% and 77.66% in response time, and 32.35% and 37.93% in battery consumption for WISDM and PAMAP2 dataset, respectively, on OnePlus 8 Pro. Overall, results in Table 3 demonstrate MobilePrune’s superiority in pruning HAR CNN models for battery usage and computational cost on today’s smartphone.

### 5.3. Ablation Studies

To demonstrate the efficacy and effectiveness of the $\ell_{0}$ sparse group lasso penalty, we performed a series of ablation studies on various DNN models. As shown in Table 4, if we only use $\ell_{0}$ norm penalty, there is no effect on a neuron or channel pruning as expected. Similarly, if we only employ the group lasso penalty, the pruned model still has more weights left. However, if we apply $\ell_{0}$ sparse group lasso, we can achieve pruned DNN models that are sparse in terms of both neurons (or channel) and weights. In addition, we compare our $\ell_{0}$ sparse group lasso model with $\ell_{1}$ sparse group lasso [59] on pruning DNN models. Table 4 shows their comparison on pruning various DNN models. More details can be found in Appendix A.3 and Appendix C. As shown in Table 4 and the results in supplementary, the $\ell_{0}$ sparse group lasso model significantly outperforms the $\ell_{1}$ sparse group lasso model in all aspects, which demonstrates its superiority in pruning DNN models.

## 6. Conclusions

In this work, we proposed a new DNN pruning method MobilePrune, which is able to generate compact DNN models that are compatible with both cuDNN and hardware acceleration. MobilePrune compress DNN models at both group and individual levels by using the novel $\ell_{0}$ sparse group lasso regularization. We further developed a global convergent optimization algorithm MobilePrune based on PALM to directly train the proposed compression models without any relaxation or approximation. Furthermore, we developed several efficient algorithms to solve the proximal operators associated with $\ell_{0}$ sparse group lasso with different grouping strategies, which is the key computation of our MobilePrune. We have performed empirical evaluations on several public benchmarks. Experimental results show that the proposed compression model outperforms existing state-of-the-art algorithms in terms of computational costs and prediction accuracy. MobilePrune has a great potential to design slim DNN models that can be deployed on dedicated hardware that uses a sparse systolic tensor array. More importantly, we deploy our system on the real android system on both Huawei P20 and OnePlus 8 Pro, and the performance of the algorithm on multiple Human Activity Recognition (HAR) tasks. The results show that MobilePrune achieves much lower energy consumption and higher pruning rate while still retaining high prediction accuracy.

There are other options to further compress the neural network models such as Neural Logic Circuits and Binary Neural Networks, which all use binary variables to represent inputs and hidden neurons. These two models are orthogonal to our methods, which means our pruning model could be adopted on Neural Logic Circuits, Binary Neural Networks and other neural network architectures designed for mobile systems. We will explore which mobile neural network could be better integrated with our network compression model in the future.

## Figures and Tables

**Table 1:** Comparison of pruned models with state-of-the-art methods on different datasets – MNIST, CIFAR-10, and Tiny-ImageNet, respectively. (We highlight our MobilePrune results and mark the best performance as blue among different methods for each model in each dataset).

Dataset	Model	Methods	Base/Pruned Accuracy (%)	Original/Remaining Parameters (Mil)	FLPOs (Mil)
MNIST		BC-GNJ [9]	98.40/98.20	267.00/28.73	28.64
		BC-GHS [9]	98.40/98.20	267.00/28.17	28.09
	LeNet-300-100	L0 [8]	-/98.60	-	69.27
		L0-sep [8]	-/98.20	-	26.64
		**MobilePrune**	**98.24/98.23**	**267.00/5.25**	**25.79**
		SBP [7]	-/99.14	-	212.80
		BC-GNJ [9]	99.10/99.00	431.00/3.88	282.87
		BC-GHS [9]	99.10/99.00	431.00/2.59	153.38
	LeNet-5	L0 [8]	-/99.10	-	1113.40
		L0-sep [8]	-/99.00	-	390.68
		**MobilePrune**	**99.12/99.11**	**431.00/2.31**	**113.50**
CIFAR-10		Original [44]	-/92.45	15.00/-	313.5
		PF [32]	-/93.40	15.00/5.4	206.3
	VGG-like	SBP [7]	92.80/92.50	15.00/-	136.0
		SBPa [7]	92.80/91.00	15.00/-	99.20
		VIBNet [39]	-/93.50	15.00/0.87	86.82
		**MobilePrune**	**92.96/92.94**	**15.00/0.60**	**77.83**
		C-OBD [40]	95.30/95.27	7.42/2.92	488.85
		C-OBS [2]	95.30/95.30	7.42/3.04	378.22
	ResNet32	Kron-OBD [40,41]	95.30/95.30	7.42/3.26	526.17
		Kron-OBS [2,41]	95.30/95.46	7.42/3.23	524.52
		EigenDamage [42]	95.30/95.28	7.42/2.99	457.46
		**MobilePrune**	**95.29**/**95.47**	**7.42/2.93**	**371.30**
		NN slimming [33]	61.56/40.05	20.12/5.83	158.62
		C-OBD [40]	61.56/47.36	20.12/4.21	481.90
		C-OBS [2]	61.56/39.80	20.12/6.55	210.05
Tiny-ImageNet	VGG-19	Kron-OBD [40,41]	61.56/44.41	20.12/4.72	298.28
		Kron-OBS [2,41]	61.56/44.54	20.12/5.26	266.43
		EigenDamage [42]	61.56/56.92	20.12/5.21	408.17
		**MobilePrune**	**61.56**/**56.27**	**20.12/4.05**	**407.37**

**Table 2:** Comparison of pruning method on the desktop with state-of-the-art methods for pruning accuracy, pruning rate and response delay on HAR datasets—WISDM, HCI-HAR, and PAMAP2, respectively. (We highlight our MobilePrune results and mark the best performance as blue among different penalties for each dataset).

Dataset	Penalty	Base/Pruned Accuracy (%)	Parameter Nonzero (%)	Parameter Remaining (%)	Node Remaining (%)	Base/Pruned Response Delay (s)	Time Saving Percentage (%)
WISDM	$l_{0}$ norm	94.72/94.79	63.36	100.00	100.00	0.38/0.39	0.00
	$l_{1}$ norm	94.30/93.84	13.58	46.26	68.16	0.38/0.24	36.84
	$l_{2}$ norm	94.61/94.54	56.28	90.46	95.12	0.38/0.35	7.89
	Group lasso	94.68/94.32	48.23	89.73	94.73	0.38/0.35	7.89
	$l_{1}$ sparse Group lasso	94.81/94.79	17.91	53.41	73.83	0.41/0.26	36.59
	**MobilePrune**	**94.97/94.65**	**9.52**	**28.03**	**52.52**	**0.50/0.17**	**66.00**
UCI-HAR	$l_{0}$ norm	91.52/91.48	88.49	100.00	100.00	0.84/0.80	4.76
	$l_{1}$ norm	90.46/90.33	81.58	98.47	99.22	0.81/0.82	0.00
	$l_{2}$ norm	91.01/90.94	88.35	100.00	100.00	0.79/0.80	0.00
	Group lasso	90.80/90.84	82.91	100.00	100.00	0.83/0.78	6.02
	$l_{1}$ sparse Group lasso	91.11/91.04	81.21	97.70	98.83	0.84/0.80	4.76
	**MobilePrune**	**90.06/89.96**	**23.00**	**46.83**	**68.75**	**1.01/0.43**	**57.43**
PAMAP2	$l_{0}$ norm	93.15/93.07	69.27	100.00	100.00	0.41/0.41	0.00
	$l_{1}$ norm	95.22/95.29	1.46	7.28	19.73	0.40/0.08	80.00
	$l_{2}$ norm	92.08/92.09	65.32	94.93	97.27	0.41/0.39	4.88
	Group lasso	93.30/93.28	61.78	100.00	100.00	0.41/0.41	0.00
	$l_{1}$ sparse Group lasso	96.87/97.20	2.67	9.72	26.17	0.40/0.10	75.00
	**MobilePrune**	**96.89/96.95**	**1.26**	**3.72**	**10.74**	**0.51/0.05**	**90.20**

**Table 3:** Comparison of pruning method on the mobile devices with other state-of-the-art pruning methods for computational cost and battery usage on HAR dataset—WISDM, HCI-HAR, and PAMAP2, respectively. (We highlight our MobilePrune results and mark the best performance as blue among different penalties for each device in each dataset).

Dataset	Device	Penalty	Base/Pruned Response Delay (s)	Time Saving Percentage (%)	Based/Pruned Device Estimated Battery Use (%/h)	Battery Saving Percentage (%)
WISDM	Huawei P20	$l_{0}$ norm	1.40/1.27	9.29	0.71/0.70	1.41
		$l_{1}$ norm	1.33/0.71	46.62	0.74/0.65	12.16
		$l_{2}$ norm	1.28/1.21	5.47	0.74/0.77	0.00
		Group lasso	1.27/1.27	0.00	0.74/0.77	0.00
		$l_{1}$ sparse Group lasso	1.25/0.81	35.20	0.74/0.68	8.11
		**MobilePrune**	**1.34/0.51**	**61.94**	**0.72/0.45**	**37.50**
	OnePlus 8 Pro	$l_{0}$ norm	0.57/0.49	14.04	0.34/0.32	5.88
		$l_{1}$ norm	0.48/0.34	29.17	0.35/0.30	14.29
		$l_{2}$ norm	0.48/0.40	16.67	0.34/0.34	0.00
		Group lasso	0.49/0.45	8.16	0.34/0.35	0.00
		$l_{1}$ sparse Group lasso	0.48/0.33	31.25	0.35/0.30	14.29
		**MobilePrune**	**0.48/0.23**	**52.08**	**0.34/0.23**	**32.35**
HCI-HAR	Huawei P20	$l_{0}$ norm	1.43/1.43	0.00	0.84/0.84	0.00
		$l_{1}$ norm	1.42/1.42	0.00	0.85/0.84	1.18
		$l_{2}$ norm	1.43/1.43	0.00	0.84/0.84	0.00
		Group lasso	1.43/1.43	0.00	0.84/0.82	2.38
		$l_{1}$ sparse Group lasso	1.42/1.41	0.70	0.85/0.82	3.53
		**MobilePrune**	**1.42/0.85**	**40.14**	**0.84/0.55**	**34.52**
	OnePlus 8 Pro	$l_{0}$ norm	0.53/0.53	0.00	0.35/0.35	0.00
		$l_{1}$ norm	0.54/0.51	5.56	0.37/0.36	2.70
		$l_{2}$ norm	0.54/0.53	1.85	0.37/0.37	0.00
		Group lasso	0.53/0.52	1.89	0.36/0.36	0.00
		$l_{1}$ sparse Group lasso	0.53/0.52	1.89	0.36/0.36	0.00
		**MobilePrune**	**0.54/0.42**	**22.22**	**0.36/0.29**	**19.44**
PAMAP2	Huawei P20	$l_{0}$ norm	2.64/2.72	0.00	0.76/0.79	0.00
		$l_{1}$ norm	2.74/0.45	83.58	0.79/0.53	32.91
		$l_{2}$ norm	2.67/2.56	4.12	0.78/0.78	0.00
		Group lasso	2.67/2.68	0.00	0.78/0.78	0.00
		$l_{1}$ sparse Group lasso	2.69/0.55	79.55	0.79/0.57	27.85
		**MobilePrune**	**2.70/0.32**	**88.15**	**0.79/0.50**	**36.71**
	OnePlus 8 Pro	$l_{0}$ norm	0.94/0.93	1.06	0.88/0.88	0.00
		$l_{1}$ norm	0.93/0.25	73.12	0.87/0.55	36.78
		$l_{2}$ norm	0.93/0.91	2.15	0.88/0.87	1.14
		Group lasso	0.94/0.95	0.00	0.89/0.89	0.00
		$l_{1}$ sparse Group lasso	0.95/0.29	69.47	0.88/0.59	32.95
		**MobilePrune**	**0.94/0.21**	**77.66**	**0.87/0.54**	**37.93**

**Table 4:** Alation studies on various network models. (We mark the best performance as blue among different penalties for each model).

Network Model	Penalty	Base/Pruned Accuracy (%)	Original/Remaining Parameters (Mil)	FLOPs	Sparsity (%)
LetNet-300	$\ell_{0}$ norm	98.24/98.46	267 K/57.45 K	143.20	21.55
	Group lasso	98.24/98.17	267 K/32.06 K	39.70	12.01
	$\ell_{1}$ sparse group lasso	98.24/98.00	267 K/15.80 K	25.88	5.93
	$\ell_{0}$ sparse group lasso	98.24/98.23	267 K/5.25 K	25.79	1.97
LetNet-5	$\ell_{0}$ norm	99.12/99.20	431 K/321.0 K	2293.0	74.48
	Group lasso	99.12/99.11	431 K/8.81 K	187.00	2.04
	$\ell_{1}$ sparse group lasso	99.12/99.03	431 K/9.98 K	183.83	2.32
	$\ell_{0}$ sparse group lasso	99.12/99.11	431 K/2.31 K	113.50	0.54
VGG-like	$\ell_{0}$ norm	92.96/93.40	15 M/3.39 M	210.94	22.6
	Group lasso	92.96/92.47	15 M/0.84 M	78.07	5.60
	$\ell_{1}$ sparse group lasso	92.96/92.90	15 M/0.61 M	134.35	4.06
	$\ell_{0}$ sparse group lasso	92.96/92.94	15 M/0.60 M	77.83	4.00
ResNet-32	$\ell_{0}$ norm	95.29/95.68	7.42 M/6.74 M	993.11	90.84
	Group lasso	95.29/95.30	7.42 M/3.03 M	373.09	40.84
	$\ell_{1}$ sparse group lasso	95.29/95.04	7.42 M/5.66 M	735.12	76.28
	$\ell_{0}$ sparse group lasso	95.29/95.47	7.42 M/2.93 M	371.30	39.49
VGG-19	$\ell_{0}$ norm	61.56/61.99	138 M/19.29 M	1519.23	13.98
	Group lasso	61.56/53.25	138 M/5.93 M	683.99	4.30
	$\ell_{1}$ sparse group lasso	61.56/53.97	138 M/0.21 M	1282.82	0.15
	$\ell_{0}$ sparse group lasso	61.56/56.27	138 M/4.05 M	407.37	2.93

**Figure A1:**
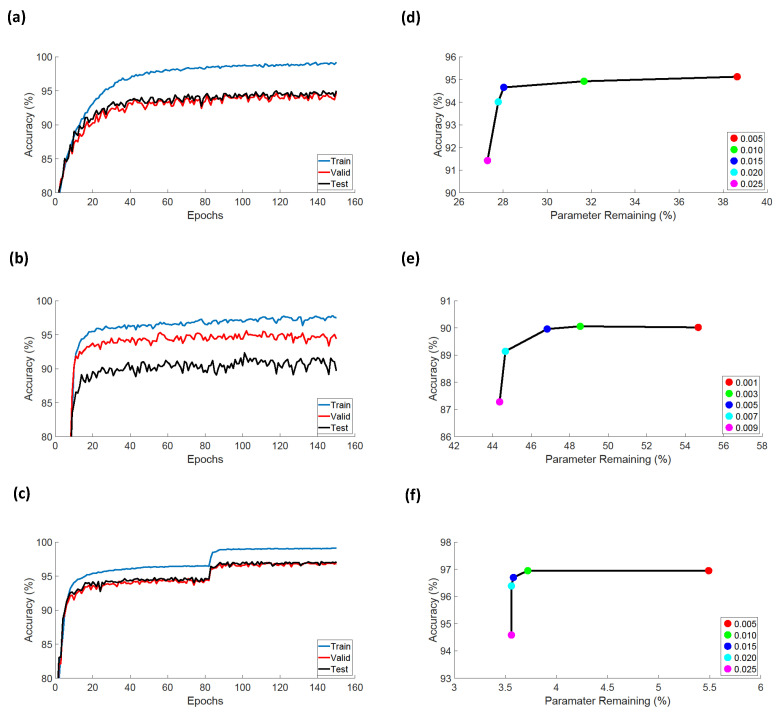
Impact of the different number of epochs (**a**–**c**) and prune thresholds (**d**–**f**) on the proposed $\ell_{0}$ sparse group lasso approach on each HAR dataset—WISDM (a,d), UCI-HAR (b,e), and PAMAP2 (c,f), respectively.

## Data Availability

We used the publicly available datasets—MNIST (http://yann.lecun.com/exdb/mnist/), CIFAR-10 (http://www.cs.toronto.edu/~kriz/cifar.html), Tiny-ImageNet (https://paperswithcode.com/dataset/tiny-imagenet), WISDM (https://archive.ics.uci.edu/ml/datasets/WISDM+Smartphone+and+Smartwatch+Activity+and+Biometrics+Dataset+), UCI-HAR (https://archive.ics.uci.edu/ml/datasets/human+activity+recognition+using+smartphones), and PAMAP2 (http://archive.ics.uci.edu/ml/datasets/pamap2+physical+activity+monitoring). Both the source codes for the proposed model results and mobile application can be found in https://github.com/yuboyubo/CNNCompressionl0 (accessed on 24 May 2022).

## Appendix A

### Appendix A.1. The Convergence Analysis of Applying PALM Algorithm to Deep Learning Models

For simplicity, we prove the convergence of the PALM algorithm on a neural network that only has fully connected layers, and the regularization is added on the weight matrix of each layer. The proof can be easily extended to DNN models with regularization added on each neuron and DNN with convolutional layers.

Given a feed-forward neural network with N−1 hidden layers, there are $d_{i}$ neurons in the *i*-th layer. Let $d_{0}$ and $d_{N}$ represent the number of neurons in the input and output layers, respectively. Therefore, the input data can be presented by $X:=\left\{x_{1},\ldots ,x_{n}\right\}\in R^{d_{0}\times n}$ and the output data can be denoted as $Y:=\left\{y_{1},\ldots ,y_{n}\right\}\in R^{d_{N}\times n}$. Let $W_{i}\in R^{d_{i}\times d_{i-1}}$ be the weight matrix between the (i−1)-th layer and the *i*-th layer. Here, in order to simplify the notation, we let $W_{i}$ absorb the bias of the *i*-th layer. We denote the collection of $W_{i}$ as $W={\left\{W_{i}\right\}}_{i=1}^{N}$. The DNN model training problem can be formulated as

(A1) $$\min_{W}:R\left(W;X,Y\right)+\sum_{i}r_{i}\left(W_{i}\right),$$

where $R\left(W;X,Y\right)=\frac{1}{n}\sum_{n}\ell \left(\Phi \left(W;x_{i}\right),y_{i}\right)$, and ℓ(·) is the loss function. $\Phi \left(W;x_{i}\right)=\sigma_{N}\left(W_{N}\sigma_{N-1}\left(...W_{2}\sigma_{1}\left(W_{1}x_{i}\right)\right)\right)$ is the DNN model with *N* layers of model parameter W and $\sigma_{i}$ is the activation function for neurons in the *i*-th layer. $r_{i}$ is the regularization function applied to $W_{i}$. We make the assumptions for our DNN model as follows:

**Assumption** **A1.**
*Suppose that the DNN model satisfies the following assumptions:*

1. *The regularization functions $r_{i},i=1,\ldots ,N$ are lower semi-continuous.*
2. *The derivatives of the loss function ℓ and all activation functions $\sigma_{i},i=1,\ldots ,N$ are bounded and Lipschitz continuous.*
3. *The loss function ℓ, activation function $\sigma_{i},\forall i$, and the regularization function $r_{i},\forall i$ are either real analytic or semi-algebraic [37], and continuous on their domains.*

**Remark** **A1.**
*The DNN model that satisfies the assumptions made in Assumption A1 could have squared, logistic, hinge, cross-entropy, or soft-max loss function and smooth activation functions, such as sigmoid, or hyperbolic tangent, and $\ell_{1}$ norm, $\ell_{2}$ norm, or $\ell_{0}$ norm regularization term, and the assumption requires the activation functions to be smooth. Then, activation function, such as rectified linear unit(ReLU), does not satisfy the requirement. However, we can use the Softplus activation function or Swish activation function to replace ReLU.*

Since R(W;X,Y) depends on weight matrices $W_{i},i=1,\ldots ,N$ between the DNN layers, we rewrite (A1) as the following format in terms of only the independent variables

(A2) $$\min_{W_{1},\ldots ,W_{N}}:\Psi \left(W\right):=H\left(W_{1},\ldots ,W_{N}\right)+\sum_{i}r_{i}\left(W_{i}\right),$$

where H is exactly the same as R in (A1), but appears in a different form. We propose Algorithm A1 to solve (A2) and prove the convergence via the following theorem. In Algorithm A1, we use the proximal operator ${\mathrm{prox}}_{t}^{\sigma }\left(x\right)$ at each step, which is defined as

(A3) $${\mathrm{prox}}_{t}^{\sigma }\left(x\right)=\underset{u}{arg min}\left\{\frac{t}{2}∥u-x∥+\sigma \left(u\right)\right\}.$$

**Theorem** **A1.**
*Suppose Assumption A1 holds. As in [37], the sequence $W^{k}=\left(W_{1}^{k},\ldots ,W_{N}^{k}\right)$ generated by Algorithm A1 converges to the critical point of (A2) if the following conditions hold:*

1. *Ψ(W) is a Kurdyka-Lojasiewicz (KL) function.*
2. *The partial gradient $\nabla_{W_{i}}H,\forall i$ is Lipschitz continuous and there exist positive constants $\underline{l_{i}},\bar{l_{i}}$ such that $c_{i}^{k}\in \left(\underline{l_{i}},\bar{l_{i}}\right),k=1,2,\ldots$.*
3. *$\nabla_{W}H\left(W_{1},\ldots ,W_{N}\right)$ has Lipschitz constant on any bounded set.*

**Proof.** For the first condition, utilizing Proposition 1 and Lemma 3–6 in [60], we can easily prove that under our assumption Ψ(W) is a KL function. For Equation (1) in the main text, it is also a KL function because $\ell_{0}$ and group lasso are semi-algebraic [60,61]. We will add the simple proof in the Appendix. According to our model assumption and Remark 1 in [62], we can show that Ψ(W) is a KL function. For the second condition, based on our model Assumption A1 and Lemma A1 provided in the following, we know that $\nabla_{W_{i}}H,\forall i$ is Lipschitz continuous. In addition, in Algorithm A1, we use backtracking strategy to estimate $L_{i}$ at each iteration, therefore, there exist $\underline{l_{i}}=L_{i}^{0}$ and $\bar{l_{i}}={\bar{L}}_{i}$, such that $c_{i}^{k}\in \left(L_{i}^{0},{\bar{L}}_{i}\right),k=1,2,\ldots$. For the last condition, based on Lemma A3 provided in the following, we have $\nabla_{W}H$$(W_{1}$, ..., $W_{N})$ is Lipschitz continuous for any bounded set.    □

**Algorithm A1** PALM Algorithm for Deep Learning Models
Initialize η>1, $L_{1}^{0},\ldots ,L_{N}^{0}$, and $W_{1}^{0},\ldots ,W_{N}^{0}$. **for** k=1,2,… **do** **for** i=1 **to** *N* **do** Find the smallest $i^{k}$ such that with $L_{i}=\eta^{i^{k}}L_{i}^{k-1}$ (A4) $$∥\nabla_{W_{i}}H\left(W_{i}^{k-1}\right)-\nabla_{W_{i}}H\left(W_{i}^{k}\right)∥\leq L_{i}∥W_{i}^{k-1}-W_{i}^{k}∥$$ Set $c_{i}^{k}=\eta^{i^{k}}L_{i}^{k-1}$ and compute (A5) $$W_{i}^{k}=prox_{c_{i}^{k}}^{r_{i}}\left\{W_{i}^{k-1}-\frac{1}{c_{i}^{k}}\nabla_{W_{i}}H\left(W_{i}^{k-1}\right)\right\}$$ **end for** **end for**

**Lemma** **A1.**
*According to Assumption A1, the derivatives of the loss function and all the activation functions used in function R in (A1) are bounded and Lipschitz continuous, then $\nabla_{W_{i}}H=\nabla_{W_{i}}R$ is also bounded and Lipschitz continuous.*

**Proof.** The partial derivative of $W_{i}$ can be written as

(A6) $$\begin{array}{cc} \nabla_{W_{i}}H & =\nabla_{W_{i}}R=\frac{\partial \ell }{\partial \Phi }\cdot \frac{\partial \Phi }{\partial \sigma_{N}}\ldots \frac{\partial \sigma_{i+1}}{\partial \sigma_{i}}\cdot \frac{\partial \sigma_{i}}{\partial W_{i}}. \end{array}$$

From Assumption A1, we know both the derivatives of the loss function $\frac{\partial \ell }{\partial \Phi }$ and the derivatives of any activation function $\frac{\partial \sigma_{i+1}}{\partial \sigma_{i}},\forall i$ are bounded and Lipschitz continuous. Based on Lemma A2 and (A6), we know the multiplication of bounded and Lipschitz continuous functions is still bounded and Lipschitz continuous. Therefore, $\nabla_{W_{i}}H$ is bounded and Lipschitz continuous.    □

**Lemma** **A2.**
*If $f_{i}:R^{N}\to R,\forall i$ is bounded $|f_{i}\left(X\right)|<M_{i},\forall i$ and Lipschitz continuous $∥f_{i}\left(X\right)-f_{i}\left(Y\right)∥\leq L_{i}∥X-Y∥$, then their multiplication $f_{1}\left(X\right)f_{2}\left(X\right)...f_{n}\left(X\right)$ is still Lipschitz continuous.*

**Proof.** We first prove that the multiplication of two bounded Lipschitz continuous functions is still Lipschitz continuous as follows.

(A7) $$\begin{array}{cc} ∥f_{1}\left(X\right)f_{2}\left(X\right)-f_{1}\left(Y\right)f_{2}\left(Y\right)∥ & =∥f_{1}\left(X\right)f_{2}\left(X\right)-f_{1}\left(X\right)f_{2}\left(Y\right)+f_{1}\left(X\right)f_{2}\left(Y\right)-f_{1}\left(Y\right)f_{2}\left(Y\right)∥\\ \; & \leq ∥f_{1}\left(X\right)f_{2}\left(X\right)-f_{1}\left(X\right)f_{2}\left(Y\right)∥+∥f_{1}\left(X\right)f_{2}\left(Y\right)-f_{1}\left(Y\right)f_{2}\left(Y\right)∥\\ \; & \leq |f_{1}\left(X\right)|L_{2}∥X-Y∥+|f_{2}\left(Y\right)|L_{1}∥X-Y∥\\ \; & \leq (M_{1}L_{2}+M_{2}L_{1})∥X-Y∥. \end{array}$$

We can then extend the above to the multiplication of multiple functions and prove the lemma.    □

**Lemma** **A3.**
*If $\nabla_{W_{i}}H,\forall i\in \left\{1,\ldots ,N\right\}$ is bounded and Lipschitz continuous, $\nabla_{W}H$ is also Lipschitz continuous.*

**Proof.** Lemma A1 has shown that $\nabla_{W_{i}}H,\forall i\in \left\{1,\ldots ,N\right\}$ is bounded and Lipschitz continuous. Here we want to show that $\nabla_{W}H$ is Lipschitz continuous on any bounded set. Define $\mathrm{vec}\left(W\right)={\left[\mathrm{vec}{\left(W_{1}\right)}^{T},\ldots ,\mathrm{vec}{\left(W_{N}\right)}^{T}\right]}^{T}$ as a vector where vec() vectorizes a matrix by stacking its columns. Specifically, there exists a constant M>0, such that for any $W,\hat{W}$

(A8) $$\begin{array}{cc} ∥\nabla_{W}H-\nabla_{\hat{W}}{H∥}^{2}= & ∥\nabla_{\mathrm{vec}(W)}H-\nabla_{\mathrm{vec}(\hat{W})}{H∥}^{2}\\ = & {∥\left[{\left(\nabla_{\mathrm{vec}(W_{1})}H\right)}^{T},\ldots ,{\left(\nabla_{\mathrm{vec}(W_{N})}H\right)}^{T}\right]-\left[{\left(\nabla_{\mathrm{vec}({\hat{W}}_{1})}H\right)}^{T},\ldots ,{\left(\nabla_{\mathrm{vec}({\hat{W}}_{N})}H\right)}^{T}\right]∥}^{2}\\ = & \sum_{i}{∥{\left(\nabla_{\mathrm{vec}(W_{i})}H\right)}^{T}-{\left(\nabla_{\mathrm{vec}({\hat{W}}_{i})}H\right)}^{T}∥}^{2}\\ \leq & \sum_{i}L_{i}^{2}{∥\mathrm{vec}\left(W_{i}\right)-\mathrm{vec}\left({\hat{W}}_{i}\right)∥}^{2}\\ \leq & L_{\max}^{2}\sum_{i}{∥\mathrm{vec}\left(W_{i}\right)-\mathrm{vec}\left({\hat{W}}_{i}\right)∥}^{2}\\ = & L_{\max}^{2}{∥W-\hat{W}∥}^{2}, \end{array}$$

where $L_{\max}$ is the largest Lipschitz constant among $L_{1},\ldots ,L_{N}$. Then, we define $M=L_{\max}$ and we have

(A9) $$\begin{array}{c} ∥\nabla_{W}H-\nabla_{\hat{W}}H∥\leq M∥W-\hat{W}∥, \end{array}$$

as desired.    □

**Theorem** **A2.**
*The sequence generated by Algorithm A1 $\left\{(W_{1}^{k},\ldots ,W_{N}^{k})\right\}$ converges to a critical point $\left(W_{1}^{*},\ldots ,W_{N}^{*}\right)$ of Equation (A2) at least in the sub-linear convergence rate, i.e. there exists some ω>0, such that*

(A10)
$$∥\left(W_{1}^{k},\ldots ,W_{N}^{k}\right)-\left(W_{1}^{*},\ldots ,W_{N}^{*}\right)∥\leq \omega k^{\frac{1-\theta }{2\theta -1}},$$

*where $\theta \in (\frac{1}{2},1)$.*

**Proof.** Based on Theorem 2 in [63] and Theorem 6.4 in [61], we can prove the above theorem.    □

### Appendix A.2. The Lemma Used in the Proof of Theorem 1

**Lemma** **A4.**
*The proximal operator associated with the Euclidean norm ∥·∥ has a closed form solution:*

(A11)
$$\underset{x}{arg min}\frac{1}{2}{∥x-y∥}^{2}+\lambda ∥x∥=\max\left(∥y∥-\lambda ,0\right)\frac{y}{∥y∥},$$

*where $x\in R^{n}$ and $y\in R^{n}$ are both n-dimensional vectors.*

This is a known result and has been used in previous study [64].

### Appendix A.3. The Algorithm for ℓ 1 Sparse Group Lasso

In Section 5.3, we conduct experiments regarding $\ell_{1}$ norm sparse group Lasso for comparison with our proposed $\ell_{0}$ sparse group lasso. Here, we elaborate the $\ell_{1}$ norm sparse group Lasso algorithm. We first consider the following proximal operator associated with $\ell_{1}$ overlapping group Lasso regularization:

(A12) $$\pi_{\lambda_{2}}^{\lambda_{1}}\left(v\right)=\underset{x\in R^{n}}{arg min}\left\{g_{\lambda_{2}}^{\lambda_{1}}\left(x\right)\equiv \frac{1}{2}{∥x-v∥}^{2}+\lambda_{1}{∥x∥}_{1}+\lambda_{2}\sum_{i=1}^{k}∥x_{G_{i}}∥\right\},$$

where the regularization coefficients, $\lambda_{1}$ and $\lambda_{2}$, are non-negative values, i=1,2,…,k, $G_{i}\subseteq \left\{1,2,\ldots ,n\right\}$ denotes the indices corresponding to the *i*-th group. According to Theorem 1 in [59], $\pi_{\lambda_{2}}^{\lambda_{1}}\left(\cdot \right)$ can be derived from the following $\pi_{0}^{\lambda_{1}}\left(\cdot \right)$ and we present this conclusion in Lemma A5.

**Lemma** **A5.**
*Let $u=\mathrm{sgn}\left(v\right)⊙\max\left(\left|v\right|-\lambda_{1},0\right)$, and*

(A13)
$$\pi_{\lambda_{2}}^{0}\left(u\right)=\underset{x\in R^{n}}{arg min}\left\{h_{\lambda_{2}}\left(x\right)\equiv \frac{1}{2}{∥x-u∥}^{2}+\lambda_{2}\sum_{i=1}^{g}∥x_{G_{i}}∥\right\}.$$

*Then, $\pi_{\lambda_{2}}^{\lambda_{1}}\left(v\right)=\pi_{\lambda_{2}}^{0}\left(u\right)$ holds.*

According to Lemma A4, it is easy to verify that given $x_{G_{i}}\cap x_{G_{j}}=\emptyset$, the optimal $x_{G_{i}}$ minimizing $h_{\lambda_{2}}\left(x\right)$ is given by

(A14) $$x_{G_{i}}=\max(∥u_{G_{i}}∥-\lambda_{2},0)\frac{u_{G_{i}}}{∥u_{G_{i}}∥},$$

where i=1,2,…,k. For the fully connected layers, we define the $\ell_{1}$ overlapping group Lasso regularizer as

(A15) $$\begin{array}{cc} \Psi \left(W\right)=\sum_{q=1}^{Q}\psi_{\lambda_{2}}^{\lambda_{1}}\left(W_{:,q}\right) & =\sum_{q=1}^{Q}\lambda_{1}∥W_{:,q}{∥}_{1}+\lambda_{2}∥W_{:,q}∥\\ & =\lambda_{1}{∥W∥}_{1}+\sum_{q=1}^{Q}\lambda_{2}∥W_{:,q}∥, \end{array}$$

where $W_{:,q}$ represents the output weights of the *q*-th neuron of *W*. According to Lemma A5, let $\hat{W}=\mathrm{sgn}\left(W\right)⊙\max\left(\left|W\right|-\lambda_{1},0\right)$, and then $\psi_{\lambda_{2}}^{\lambda_{1}}\left(W_{:,q}\right)$ can be reduced to $\psi_{\lambda_{2}}^{0}\left({\hat{W}}_{:,q}\right)$, which can be solved via (A14) as follows.

(A16) $$W_{:,q}=\max(∥{\hat{W}}_{:,q}∥-\lambda_{2},0)\frac{{\hat{W}}_{:,q}}{∥{\hat{W}}_{:,q}∥}$$

For the convolutional layers, we denote the $\ell_{1}$ overlapping group Lasso regularizer as

(A17) $$\begin{array}{cc} \Phi \left(T\right)=\sum_{c=1}^{N_{c}}\phi_{\beta_{b}}^{\beta_{a}}\left(T_{:,c,:,:}\right) & =\sum_{c=1}^{N_{c}}\beta_{a}∥T_{:,c,:,:}{∥}_{1}+\beta_{b}\sum_{h=1}^{N_{h}}\sum_{w=1}^{N_{w}}∥T_{:,c,h,w}∥\\ \; & =\beta_{a}{∥T∥}_{1}+\sum_{c=1}^{N_{c}}\sum_{h=1}^{N_{h}}\sum_{w=1}^{N_{w}}\beta_{b}∥T_{:,c,h,w}∥, \end{array}$$

Likewise, let ${\hat{T}}_{:,c,:,:}=\mathrm{sgn}\left(T_{:,c,:,:}\right)⊙\max\left(|T_{:,c,:,:}|-\beta_{a},0\right)$, and then $\phi_{\beta_{b}}^{\beta_{a}}\left(T_{:,c,:,:}\right)$ can be reduced to $\phi_{\beta_{b}}^{0}\left({\hat{T}}_{:,c,:,:}\right)$, which can be solved via (A14) as follows.

(A18) $$T_{:,c,h,w}=\max(∥{\hat{T}}_{:,c,h,w}∥-\beta_{b},0)\frac{{\hat{T}}_{:,c,h,w}}{∥{\hat{T}}_{:,c,h,w}∥}$$

By incorporating (A15) and (A17) into our model, the $\ell_{1}$ norm sparse group Lasso regularized problem can be formulated as

(A19) $$\min_{W}:L\left(W;X,Y\right)+\sum_{i=1}^{N_{f}}\sum_{q=1}^{Q}\psi_{\lambda_{2}}^{\lambda_{1}}\left(W_{:,q}^{\left(i\right)}\right)+\sum_{j=1}^{N_{l}}\sum_{c=1}^{N_{c}}\phi_{\beta_{b}}^{\beta_{a}}\left(T_{:,c,:,:}^{\left(j\right)}\right).$$

We elaborate the DNN_PALM algorithm associated with $\ell_{1}$ norm sparse group lasso in Algorithm A2, in which the partial derivatives are denoted as $H\left(W_{:,q}^{\left(i\right)}\right)=\nabla_{W_{:,q}^{\left(i\right)}}L\left(W_{:,q}^{\left(i\right)}\right)$ and $F\left(T_{:,c,h,w}^{j}\right)=\nabla_{T_{:,c,h,w}^{j}}L\left(T_{:,c,h,w}^{j}\right)$ for fully connected layers and convolutional layers, respectively.

**Algorithm A2** DNN_PALM Algorithm for $\ell_{1}$ norm Group Lasso
Initialize μ>1, $L_{i}^{0}>0,{\left(W^{\left(i\right)}\right)}^{0},\forall i$ and $L_{j}^{0}>0,{\left(T^{\left(j\right)}\right)}^{0},\forall j$. **for**k=1,2,…**do** **for** i=1 **to** $N_{f}$ **do** Let $\omega^{k-1}={\left(W_{:,q}^{\left(i\right)}\right)}^{k-1}$ and $\omega^{k}={\left(W_{:,q}^{\left(i\right)}\right)}^{k},\;\forall q$. Find the smallest $L=\mu^{b}L_{i}^{k-1},b\in N$, such that $∥H\left(\omega^{k-1}\right)-H\left(\omega^{k}\right)∥\leq L∥\omega^{k-1}-\omega^{k}∥$, where $\omega^{k}$ is computed via (A16). **end for** **for** j=1 **to** $N_{l}$ **do** **for** c=1 **to** $N_{c}$ **do** Let $\gamma^{k-1}={\left(T_{:,c,h,w}^{j}\right)}^{k-1}$ and $\gamma^{k}={\left(T_{:,c,h,w}^{j}\right)}^{k},\;\forall h,w$. Find the smallest $L=\mu^{b}L_{j}^{k-1},b\in N$, such that $∥F\left(\gamma^{k-1}\right)-F\left(\gamma^{k}\right)∥\leq L∥\gamma^{k-1}-\gamma^{k}∥$, where $\gamma^{k}$ is computed via (A18). **end for** **end for** **end for**

## Appendix B

### Appendix B.1. Iterative Method

Iterative pruning [4] is another effective method for obtaining a sparse network while maintaining high accuracy. As iterative pruning is orthogonal to our method, we can couple the two methods to obtain even better performance per number of parameters used; specifically, we replace the usual weight decay regularizer used in [4] with our $\ell_{0}$ sparse group lasso regularizer. In practice, we find that, empirically, the iterative method is able to achieve better performance. All results reported in the paper are from the iterative method.

### Appendix B.2. Hyper-Parameter Settings

In our experiments, all the baseline models were trained from scratch via stochastic gradient decent(SGD) with a momentum of 0.9. We trained the networks for 150 epochs on MNIST and 400 epochs on CIFAR-10 and Tiny-ImageNet with an initial learning rate of 0.1 and weight decay of 5e-4. The learning rate is decayed by a factor of 10 at 50, 100 on MNIST and at 100, 200 on CIFAR-10 and Tiny-ImageNet, respectively.

The experimental settings regarding hyper-parameters for all DNN models we used in the paper are summarized in Table A1. We employ iterative pruning strategy to prune all the models. Namely, the pruning process and the retraining process are performed alternately.

**Table A1:** List of hyper-parameters and their values(“-” denotes “not applicable”).

Hyper-Parameter	LeNet300	LeNet5	VGG-Like	ResNet-32	VGG-19	Description
learning rate	1 × 10^−3^	1 × 10^−3^	1 × 10^−3^	1 × 10^−3^	1 × 10^−3^	The learning rate used in retraining process
gradient momentum	0.9	0.9	0.9	0.9	0.9	The gradient momentum used in retraining process
weight decay	1 × 10^−4^	1 × 10^−5^	5 × 10^−4^	1 × 10^−4^	1 × 10^−4^	The weight decay factor used in retraining process
minibatch size	1 × 10^2^	6 × 10^2^	1 × 10^3^	3 × 10^2^	4 × 10^2^	The number of training samples over which each SGD update is computed during the retraining process
$\ell_{0}$ norm factor	4 × 10^−4^	2 × 10^−4^	1 × 10^−6^	1 × 10^−8^	1 × 10^−10^	The shrinkage coefficient for $\ell_{0}$ norm regularization
channel factor	-	1 × 10^−3^	1 × 10^−3^–1 × 10^−2^ ^1^	5 × 10^−2^	5 × 10^−2^	The shrinkage coefficient of channels for group Lasso
neuron factor	2 × 10^−4^	2 × 10^−4^	1 × 10^−4^	0	1 × 10^−2^	The shrinkage coefficient of neurons for group Lasso
filter size factor	-	1 × 10^−3^	1 × 10^−4^	1 × 10^−4^	1 × 10^−4^	The shrinkage coefficient of filter shapes for group Lasso
pruning frequency (epochs/minibatches)	10	10	1	2	1	No. of epochs(LeNet)/minibatches(VGGNet/ResNet) for pruning before retraining
retraining epochs	30	30	20	30	15	The number of retraining epochs after pruning
iterations	74	102	63	2	66	The number of iterations for obtaining the final results

^1^ On VGG-like, the channel factor is adaptive and it is increased by 0.001 if its cross-entropy loss is not greater than the loss before performing pruning for the current mini-batch. Its range is [0.001,0.01].

## Appendix C

### Appendix C.1. Computational Efficiency

We want to mention that our main focus here is to compress DNN models via $\ell_{0}$ sparse group lasso. Our main contribution is to solve the corresponding optimizations. Speed is not our primary focus. However, we still provide the run time of our methods as a reference. We compared the run time of our MobilePrune with the baseline methods (original methods without pruning involved), and we found the ratio of the run time of MobilePrune to the run time of the baseline method is around 5 on average on the same machine.

### Appendix C.2. Results about the SSL

**Table A2:** Results about learning filter shapes in LeNet-5. (We highlight our MobilePrune results).

Method	Base/Pruned Accuracy (%)	Filter Size	Remaining Filters	Remaining Parameters	FLOPs (K)
Baseline	-	25–500	20–50	500–25,000	2464
SSL [24]	99.10/99.00	7–14	1–50	-	63.82
**MobilePrune**	**99.12/99.03**	**14–9**	**4–16**	**46–26**	**51.21**

### Appendix C.3. Additional Ablation Studies

In this section, we perform ablation studies to compare DNN models regularized by the proposed $\ell_{0}$ sparse group lasso and other DNN models that are regularized by its individual components. Specifically, we compare DNN models regularized by the proposed $\ell_{0}$ sparse group lasso with DNN models regularized by $\ell_{0}$ norm penalty (set group Lasso penalty to 0) and DNN models regularized by group Lasso penalty (set $\ell_{0}$ norm penalty to 0), respectively. For fair comparison, for all regularized DNN models, we use the same hyper-parameter setting.

From Table A3 to Table A6, we observe that $\ell_{0}$ norm penalty has no effect on structured pruning as expected and group Lasso penalty can effectively remove redundant structure components. Furthermore, the combination of $\ell_{0}$ norm and group Lasso (our proposed $\ell_{0}$ sparse group lasso penalty) can yield sparser models at both structure level and individual weight level. Notably, $\ell_{0}$ norm can help group Lasso to remove more redundant structure components. Therefore, better acceleration in terms of FLOPs can be obtained by applying our proposed $\ell_{0}$ sparse group lasso penalty. We want to mention that when we compute FLOPs, we do not take the individual weight sparsity into account. However, based on [11,65], lower FLOPs could be achieved by unitizing the sparsity on weight level on dedicated architectures.

**Table A3:** Alation studies on LetNet-5 (Architecture: 20-50-800-500).

Penalty	Base/Pruned Accuracy (%)	Original/Remaining Parameters (K)	Pruned Architecture	Filter Size	FLOPs (K)	Sparsity (%)
$\ell_{0}$ norm	99.12/99.20	431/321.00	20-50-800-500	25–500	2293.0	74.48
Group Lasso	99.12/99.11	431/8.81	4-19-301-29	25–99	187.00	2.04
$\ell_{1}$ Group Lasso	99.12/99.03	431/9.98	4-17-271-82	23–99	183.83	2.32
$\ell_{0}$ sparse group lasso	99.12/99.11	431/2.31	5-14-151-57	16–65	113.50	1.97

**Table A4:** Alation studies on VGG-like.

Penalty	Base/Pruned Accuracy (%)	Original/Remaining Parameters (Mil)	Pruned Architecture	FLOPs (Mil)
$\ell_{0}$ norm	92.96/93.40	15/3.39	18-43-92-99-229-240-246-507-504-486-241-114-428-168	210.94
Group Lasso	92.96/92.47	15/0.84	17-43-89-99-213-162-93-42-32-28-8-5-429-168	78.07
$\ell_{1}$ Group Lasso	92.96/92.90	15/0.61	17-43-92-99-229-240-246-323-148-111-41-39-159-161	134.35
$\ell_{0}$ sparse group lasso	92.96/92.94	15/0.60	17-43-87-99-201-185-80-37-27-25-9-4-368-167	77.83

**Table A5:** Alation studies on ResNet-32.

Penalty	Base/Pruned Accuracy (%)	Original/Remaining Parameters (Mil)	FLOPs (Mil)	Sparsity (%)
$\ell_{0}$ norm	95.29/95.68	7.42/6.74	993.11	90.84
Group Lasso	95.29/95.30	7.42/3.43	393.09	45.95
$\ell_{1}$ Group Lasso	95.29/95.04	7.42/5.66	735.12	76.28
$\ell_{0}$ sparse group lasso	95.29/95.47	7.42/2.93	371.30	39.49

**Table A6:** Alation studies on VGG19.

Penalty	Test Accuracy (%)	Remaining Parameters (Mil)	Pruned Architecture	FLOPs (Mil)
Baseline	61.56	20.12	64-64-128-128-256-256-256-256-512-512-512-512-512-512-512-512	1592.53
$\ell_{0}$ norm	61.99	19.29	45-64-114-128-256-256-256-256-512-511-512-509-512-512-512-512	1519.23
Group Lasso	53.25	5.93	23-61-80-128-122-114-164-253-255-322-412-462-23-93-129-512	683.99
$\ell_{1}$ Group Lasso	53.97	0.21	29-64-109-128-254-246-254-256-510-509-509-509-512-512-484-512	1282.82
$\ell_{0}$ sparse group lasso	56.27	4.05	19-48-57-102-79-83-100-179-219-273-317-341-256-158-116-512	407.37

### Appendix C.4. Additional Comparison between ℓ 0 Sparse Group Lasso and ℓ 1 Norm Sparse Group Lasso

In addition, we compare the proposed $\ell_{0}$ sparse group lasso with $\ell_{1}$ norm group Lasso. The algorithm for DNN models with $\ell_{1}$ norm group Lasso penalty is introduced in Algorithm A2. For hyper-parameter setting, we use the same parameters as $\ell_{0}$ sparse group lasso penalty (as shown in Table A1) except the parameter for $\ell_{1}$ norm. We search the parameter of $\ell_{1}$ norm in [0.0001,0.01] and report the best results in terms of the pruned test accuracy.

From Table A3 to Table A6, we find that $\ell_{0}$ sparse group lasso penalized models outperform $\ell_{1}$ sparse group Lasso penalized models in terms of test accuracy and FLOPs. For VGG19 model (Table A6), $\ell_{1}$ sparse group Lasso penalized model can achieve the fewest number of parameters, but the pruned test accuracy and FLOPs are much worse than the $\ell_{0}$ sparse group lasso penalized model.

### Appendix C.5. The Effect of the Coefficient of ℓ 0 Norm Regularizer

In our proposed $\ell_{0}$ sparse group lasso, $\ell_{0}$ norm regularizer plays an important role of facilitating pruning networks effectively and efficiently, which has been shown through the results in ablation studies with and without $\ell_{0}$ norm penalty. We further explore the effect of the strength of the $\ell_{0}$ norm coefficient on the pruning performance. We vary the shrinkage strength for $\ell_{0}$ norm penalty by a factor of 10 while keeping the other settings fixed.

As can be seen from Table A7 and Table A8, the larger the $\ell_{0}$ norm coefficient is, the more parameters are pruned as expected. Additionally, there is a trade-off between the shrinkage coefficients for $\ell_{0}$ norm penalty and group Lasso penalty, which depends on the practical demand.

**Table A7:** The effect of the coefficient of $\ell_{0}$ norm penalty on VGG-like.

$\ell_{0}$ Penalty Coefficient	Base/Pruned Accuracy (%)	Original/Remaining Parameters (Mil)	Pruned Architecture	FLOPs (Mil)
1 × 10^−4^	92.96/89.77	15/0.06	17-43-83-99-161-105-57-28-24-15-11-4-104-157	56.43
1 × 10^−5^	92.96/92.19	15/0.30	16-43-85-99-171-155-75-33-23-18-10-3-264-167	66.82
1 × 10^−6^	92.96/92.94	15/0.60	17-43-87-99-201-185-80-37-27-25-9-4-368-167	77.83
1 × 10^−7^	92.96/92.54	15/0.74	17-43-87-99-213-188-91-40-26-27-9-4-400-168	81.64

**Table A8:** The effect of the coefficient of $\ell_{0}$ norm penalty on ResNet-32.

$\ell_{0}$ Penalty Coefficient	Base/Pruned Accuracy (%)	Original/Remaining Parameters (Mil)	FLOPs (Mil)	Sparsity
1 × 10^−6^	95.29/95.11	7.42/2.06	330.90	27.76
1 × 10^−7^	95.29/95.33	7.42/2.72	369.36	36.66
1 × 10^−8^	95.29/95.47	7.42/2.93	77.83	39.49
1 × 10^−9^	95.29/95.44	7.42/3.02	372.98	40.70

## Appendix D

### Appendix D.1. Har Dataset Description

#### Appendix D.1.1. Wisdm Dataset

The WISDM dataset [49], publicly available in [50], includes six activities (walking, jogging, walking upstairs, walking downstairs, sitting, and standing) that contain 3D (x,y,z) raw signals collected from the smartphone’s accelerometer at a sampling rate of 20Hz. The total number of participants involved in the experiment is 36. These participants performed certain daily activities with an Android phone in their front leg pockets. This dataset has a total of 1,098,209 samples and each sample consists of a timestamp, a user ID, an activity ID, and the acceleration (x,y,z) raw data. Here for this dataset, 3 features are used—the gravitational acceleration (x,y,z) toward the center of the Earth. A sliding window approach with a window size of 80 readings (4 seconds) is used for segmenting the sequences with a 50% overlapping.

#### Appendix D.1.2. UCI-HAR Dataset

The UCI-HAR [51] dataset, publicly available in [52], includes six activities (walking, walking upstairs, walking downstairs, sitting, standing, and jogging) that contains 3D (x,y,z) raw signals extracted from the accelerometer and gyroscope of a smartphone at a constant rate of 50 Hz strapped to the waist of a subject. These raw signals were applying a noise filter to remove the noise first and then sampled in fixed-width sliding windows of 2.56 s (128 readings). This dataset was collected from a group of 30 volunteers. And all volunteers were instructed to follow an activity protocol and wore a Samsung Galaxy S II smartphone on their waist. The dataset includes a total of 10,299 samples including 7352 training samples (71.39%) and 2947 testing samples (28.61%). The dimension for each sample is 128 readings × number of features with a 50% overlapping. Here for this dataset, 9 features are used—the acceleration signals (x,y,z) collected by the smartphone accelerometer in standard gravity units, the body acceleration signals (x,y,z) obtained by subtracting the gravity from the total acceleration, and the angular velocity vector (x,y,z) measured by the smartphone gyroscope.

#### Appendix D.1.3. PAMAP2 Dataset

The PAMAP2 dataset [53,54], publicly available in [55], contains data of different physical activities, performed by 9 subjects wearing 3 inertial measurement units and a heart rate monitor with a sampling rate of 100 Hz. According to the dataset’s protocol, there are 12 physical activities—lying, sitting, standing, walking, running, cycling, nordic walking, ascending stairs, descending stairs, vacuum cleaning, and rope jumping. All the collected data above include two 3-axis accelerometer data, 3-axis gyroscope data, 3-axis magnetometer data, 3-axis orientation data, and temperature. This dataset has a total of 3,850,505 samples and each sample has a timestamp, a user id, an activity id, and the corresponding features. Here we pick 40 features listed by the dataset. Similar to the WISDM dataset, a sliding window approach with a window size of 128 readings (1.28 s) is used for segmenting the sequences with a 50% overlapping.

### Appendix D.2. 1D CNN Model

In general, sensor data, such as accelerometers and gyroscopes, can be classified as time-series data. We can encode these time-series data as images to allow machines to recognize human behavior virtually. Inspired by the recent successes of deep learning techniques in computer vision, we convert sequence data into image data according to Gramain Angular Field transform algorithm [66] to obtain a one-dimensional convolutional neural network (1D CNN) model. The benefit of using CNNs for sequence classification is that we can learn from the raw time series data directly, and this method does not require domain expertise to manually engineer input features. This 1D CNN model can learn an internal representation of the time series data and could achieve good performance to models that fit on the version of the dataset with engineered features.

Similar to the general CNN model, the convolutional layer that uses the convolution kernel for the input data is the most essential part in CNN. The convolution kernel works as a filter and is activated by a non-linear activation function. In this paper, a sequential model with a PyTorch backend is built via Google Colab [56] and 5 consecutive 1D convolutional layers with 128 neurons each are selected. Each layer uses a ReLU activation function. In order to compress this CNN model, we cannot add a 1D max-pooling because of the random down-sampling. For the WISDM and PAMAP2 datasets, 10 convolution kernels are used for each layer. For the UCI-HAR dataset, 5 convolution kernels are used. All these values are selected based on tons of experiments. In addition, for the sake of efficiency, we select the number of epochs to be 150 for all three datasets during the training stage.

### Appendix D.3. Data Pre-Processing

In order to provide a certain data dimension and improve the performance of the proposed 1D CNN Model, the above collected raw data need to be pre-processed as the following.

#### Appendix D.3.1. Re-Scaling and Standardization

If we use the above datasets’ raw data directly to train our model, the final results may cause training bias because of those large values. In order to remove such bias, standardizing a dataset is necessary. Standardizing a dataset involves re-scaling the distribution of the values for each channel such that the mean is 0 and the standard deviation is 1, as shown in Equation (A20):

(A20) $$\begin{array}{c} X_{ij}=\frac{X_{ij}-mean\left(X_{i}\right)}{std(X_{i})} \end{array}$$

where i=1,2,…,n and *n* denotes the number of channels, j=1,2,…,m and *m* denotes the number of elements in each channel.

#### Appendix D.3.2. Segmentation

As mentioned above, the input to the model consists of a data sequence extracted from the raw sensor data. The data were recorded continuously in the data collection process. In order to preserve the temporal relationship between the collected data points and their corresponding activity, a sliding window approach is used to segment the collected data points. For the HCI-HAR and PAMAP2 datasets, a fixed-length of 128 sliding windows with an overlapping rate of 50% is applied. For the WISDM dataset, a fixed-length of 80 sliding windows with an overlapping rate of 50% is applied. After segmenting the raw data, we obtain 27,455 samples for the WISDM dataset, 10,299 samples for the UCI-HAR dataset, and 30,356 samples for the PAMAP2 dataset. Next, we select 80% data samples randomly from WISDM and PAMAP2 datasets as the training samples while the remaining 20% data samples are the testing samples. For the UCI-HAR dataset, it is already split.

#### Appendix D.3.3. K-Fold Cross-Validation

In order to improve the proposed model’s final performance, k-fold cross-validation is used after the above segmentation step. The principle of the k-fold cross-validation method is to split the input samples as the number of *k* groups. It can lead to a less biased or less positive assessment of the ability of the model than other methods [67]. All the training data samples are considered for both training and validation in a k-fold cross-validation approach. First, we divide the training data samples into *k* equal subsets. Then, we pick one subset as the validation set and the remaining k−1 subsets as the training set. There are *k* different ways to select the validation set, and therefore we have *k* different pairs of testing and validation datasets. In this paper, we choose k=5 and evaluate all the 5 different pairs of testing and validation datasets for our proposed method and all state-of-the-art pruning methods. The final training and validation dataset will be selected according to the final performance with the testing data set.

### Appendix D.4. Hyper-Parameters Tuning

Hyper-parameters have a great impact on the deep learning model performance. In the following context, we will present how to select the training subset, validation subset based on the 5-fold cross-validation before the training stage, how to pick the learning rate during the training stage, how to select the model by the epochs during the training stage, and how to pick pruning threshold to compress the final model. The experiments are implemented on all three datasets and the model performance is evaluated by varying several model parameters.

#### Appendix D.4.1. Cross-Validation Tuning

In order to improve the proposed model’s final performance, k-fold cross-validation is used after segmenting the input samples. This approach can lead to a less biased or less positive assessment of the ability of the model than other methods [67]. Table A9 shows the results on the test set corresponding to different validation set choices. The pruned accuracy results are obtained when we use the fold numbers 4, 5, and 1 as the validation set based on both pruned accuracy and the nonzero parameters’ percent for the WISDM, UCI-HAR, PAMAP2 datasets, respectively.

#### Appendix D.4.2. Learning Rate Tuning

The learning rate is a hyper-parameter that controls how much to change the model in response to the estimated error each time the model weights are updated [68]. Table A9 demonstrates the experiment results of different learning-rate settings. For the WISDM dataset, we observe that the best performance of pruned accuracy and parameter remaining percentage is achieved when the learning rate equals $1.5\times 10^{-4}$. For the UCI-HAR dataset, when the learning rate is $2\times 10^{-4}$, we obtain the best results of pruned accuracy and parameter remaining percentage. For the PAMAP2 dataset, the best-pruned accuracy is achieved when the learning rate is $1.5\times 1.0^{-4}$. To make the experiment settings consistent and comparable, we set the learning rate to be $1.0\times 10^{-4}$ for all three datasets.

**Table A9:** Impact of different cross-validation fold numbers and learning rates on the proposed $\ell_{0}$ sparse group lasso approach on each HAR dataset—WISDM, UCI-HAR, and PAMAP2, respectively. (We highlight our selection in both fold number and learning rate for each dataset).

Dataset	Type	Value	Base/Pruned Accuracy (%)	Parameter Nonzero (%)	Parameter Remaining (%)	Node Remaining (%)
WISDM	Fold Number	1	93.52/92.68	11.64	32.49	57.42
		2	94.88/93.70	10.03	30.35	55.08
		3	94.45/93.48	9.45	27.97	52.13
		**4**	**94.97/94.65**	**9.52**	**28.03**	**53.52**
		5	93.52/92.68	11.64	32.49	57.42
	Learning Rate	1.0 $\times 10^{-5}$	89.55/86.72	27.09	93.50	96.68
		5.0 $\times 10^{-5}$	92.93/84.36	9.41	40.44	64.06
		**1.0 $\times 10^{-4}$**	**94.97/94.65**	**27.09**	**28.03**	**53.52**
		1.5 $\times 10^{-4}$	94.96/94.88	10.38	27.26	52.54
		1.0 $\times 10^{-4}$	94.65/94.57	10.54	32.38	56.84
UCI-HAR	Fold Number	1	78.42/78.08	15.53	31.99	56.64
		2	89.89/89.28	32.49	64.29	80.27
		3	79.13/79.37	16.02	32.25	56.84
		4	78.22/78.22	18.69	40.02	63.48
		**5**	**90.06/89.96**	**23.00**	**46.83**	**68.75**
	Learning Rate	1.0 $\times 10^{-5}$	85.27/85.51	77.98	94.66	97.27
		5.0 $\times 10^{-5}$	89.38/89.24	16.69	85.77	92.58
		**1.0 $\times 10^{-4}$**	**90.06/89.96**	**23.00**	**46.83**	**68.75**
		1.5 $\times 10^{-4}$	90.94/90.91	16.69	31.04	56.45
		2.0 $\times 10^{-4}$	90.40/90.43	13.24	29.10	54.10
PAMAP2	Fold Number	**1**	**96.89/96.95**	**1.26**	**3.72**	**10.74**
		2	92.29/92.28	1.27	3.15	10.35
		3	96.49/96.28	1.81	4.74	14.84
		4	95.08/94.99	1.20	3.42	10.55
		5	94.81/94.81	1.46	3.71	11.52
	Learning Rate	1.0 $\times 10^{-5}$	93.63/85.80	7.93	28.61	49.22
		5.0 $\times 10^{-5}$	94.25/93.89	3.90	11.81	28.32
		**1.0 $\times 10^{-4}$**	**96.89/96.95**	**1.26**	**3.72**	**10.74**
		1.5 $\times 10^{-4}$	96.57/96.62	1.12	2.36	7.62
		2.0 $\times 10^{-4}$	94.89/94.99	0.68	2.02	7.62

#### Appendix D.4.3. Number of Epochs Tuning

The number of epochs is a hyper-parameter that defines the number of times that the learning algorithm will work through the entire training dataset. Figure A1a–c show the training accuracy, validation accuracy, and testing accuracy versus the number of epochs for WISDM, UCI-HAR, and PAMAP2 datasets, respectively. For all three datasets, the number of epochs is 150; however, we only pick the epoch number based on the highest accuracy of the validation set for each dataset. The validation dataset is different from the test dataset, but is instead used to give an unbiased estimate of our final model. Based on the final results, the highest validation accuracy occurs at 150, 102, 113 epochs for WISDM, UCI-HAR, and PAMAP2 datasets, respectively.

#### Appendix D.4.4. Prune Threshold Tuning

For all the pruning methods including $\ell_{0}$ sparse group lasso, after pruning, the weights cannot be exact zero due to the binary bits computation. Therefore, if weight is less than a costumed prune threshold, we set the weight to be zero in those pruned models. Figure A1d–f shows the experiment results of different prune threshold settings for WISDM, UCI-HAR, and PAMAP2 datasets, respectively. For the WISDM dataset, we observe that the best performance of pruned accuracy and parameter remaining percentage is achieved when the threshold equals 0.015. For the UCI-HAR dataset, when the pruned threshold is 0.005, we obtain the best results of pruned accuracy and parameter remaining percentage. For the PAMAP2 dataset, the best-pruned accuracy is achieved when the pruned threshold is 0.01.
